# Dosage constraint of the ribosome-associated molecular chaperone drives the evolution and fates of its duplicates in bacteria

**DOI:** 10.1128/mbio.01994-24

**Published:** 2024-10-07

**Authors:** Tianyu Wan, Li Zhuo, Zhuo Pan, Rui-yun Chen, Han Ma, Ying Cao, Jianing Wang, Jing-jing Wang, Wei-feng Hu, Ya-jun Lai, Muhammad Hayat, Yue-zhong Li

**Affiliations:** 1 State Key Laboratory of Microbial Technology, Institute of Microbial Technology, Shandong University, Qingdao, China; 2 Shenzhen Research Institute, Shandong University, Shenzhen, China; 3 Suzhou Research Institute, Shandong University, Suzhou, China; University of Pittsburgh School of Medicine, Pittsburgh, Pennsylvania, USA

**Keywords:** trigger factor, gene duplication, dosage constraint, ribosome binding, holdase

## Abstract

Gene duplication events happen prevalently during evolution, and the mechanisms governing the loss or retention of duplicated genes are mostly elusive. Our genome scanning analysis revealed that trigger factor (TF), the one and only bacterial ribosome-associated molecular chaperone, is singly copied in virtually every bacterium except for a very few that possess two or more copies. However, even in these exceptions, only one complete TF copy exists, while other homologs lack the N-terminal domain that contains the conserved ribosome binding site (RBS) motif. Consistently, we demonstrated that the overproduction of the N-terminal complete TF proteins is detrimental to the cell, which can be rescued by removing the N-terminal domain. Our findings also indicated that TF overproduction leads to a decrease in protein productivity and profile changes in proteome due to its characteristic ribosome binding and holdase activities. Additionally, these N-terminal deficient TF homologs in bacteria with multiple TF homologs partition the function of TF via subfunctionalization. Our results revealed that TF is subjected to a dosage constraint that originates from its own intrinsic functions, which may drive the evolution and fates of duplicated TFs in bacteria.

**IMPORTANCE:**

Gene duplication events presumably occur in *tig*, which encodes the ribosome-associated molecular chaperone trigger factor (TF). However, TF is singly copied in virtually every bacterium, and these exceptions with multiple TF homologs always retain only one complete copy while other homologs lack the N-terminal domain. Here, we reveal the manner and mechanism underlying the evolution and fates of TF duplicates in bacteria. We discovered that the mutation-to-loss or retention-to-sub/neofunctionalization of TF duplicates is associated with the dosage constraint of N-terminal complete TF. The dosage constraint of TF is attributed to its characteristic ribosome binding and substrate-holding activities, causing a decrease in protein productivity and profile changes in cellular proteome.

## INTRODUCTION

Gene duplication is prevalent across diverse organisms (1), and the majority of duplicated genes become nonfunctional by the process of “nonfunctionalization” and are ultimately eliminated (2). This process is typically attributed to functional redundancy (3, 4), but is poorly understood. A significant fraction of retained duplicates are believed to undergo neofunctionalization, subfunctionalization, dosage ampliﬁcation, back-up compensation, and structural and functional entanglement (5). Deciphering the underlying mechanisms that govern the loss or retention of duplicated genes is crucial for determining the driving forces that underly evolution and the fates of duplicated genes.

Trigger factor (TF), encoded by the *tig* gene, is the only bacterial ribosome-associated molecular chaperone that binds to the 50S subunit of the ribosome and locates at the rim of the ribosomal tunnel exit for the primary refolding of nascent peptide chains (6). The chaperone is responsible for refolding and stabilizing approximately 70% of total bacterial proteins in an ATP-independent manner (7–9). TF is a modular protein that contains an N-terminal domain, an intermediate peptidylprolyl isomerase (PPIase) domain, and a C-terminal domain (8, 10). The N-terminal domain contains a conserved ribosome binding site (RBS), GFR×G××P (11). In addition to ribosome binding ability, the N-terminal domain is associated with the chaperone activity of TF, as defects in this fragment cause a loss of aggregation-inhibiting activity *in vitro* (12). The C-terminal domain is folded to the middle of the spatial structure and spatially forms a finger-like structure which, together with the N-terminal domain, creates a hydrophobic cavity to isolate the nascent peptide chain and shield their hydrophobic regions that are prone to misaggregation (8, 11, 13). The C-terminal domain plays an essential role in substrate binding activity (14–16). The middle PPIase domain of TF belongs to the FKBP (FK506-binding protein) type (10, 17), and exerts cis-trans isomerization activity on the proline imidic peptide bonds in oligopeptides (10, 18). Although the PPIase domain is relatively conserved (19), PPIase activity does not seem essential for the chaperone activity of TF (12, 20). This chaperone is generally assumed to be a holdase and plays a passive role in protein folding (15). TF exists in cells as a monomer or a dimer, and monomeric TF engages ribosome binding and acts early during the folding process (21, 22). In contrast, dimeric TF holds unsettled peptide chains after being released from the ribosome to prevent aggregation before turning to downstream chaperones, such as DnaK or GroEL, for further folding and maturation (22, 23).

Deletion of *tig* typically does not impact cellular growth, probably due to functional overlap with DnaK or GroEL (24–28). However, the overproduction of *tig* leads to abnormal filamentation of *Escherichia coli* cells (29). Although the physiological concentration of TF is relatively high in bacterial cells as it exceeds the concentration of ribosomes by more than twofold in *E. coli* (30), analysis of the published transcriptomes revealed that the transcription of *tig* is significantly lower than that of *dnaK* or *groEL* in *E. coli* (31), *Bacillus subtilis* (32), and *Myxococcus xanthus* (33). A similar dosage constraint was also observed in *in vitro* experiments: low concentration of TF proteins (10 µM or lower) could reverse the activity of predenatured glyceraldehyde-3-phosphate dehydrogenase (GAPDH) (34, 35); however, high TF concentration (four times greater than those of the substrate) restrained the recovery by forming stable intermediate TF-substrate complexes (12, 15, 35, 36). These *in vivo* and *in vitro* results suggested that a quantitative limitation is placed on the intracellular functions of the TF chaperone via an unknown mechanism.

In this paper, we report the scanning of TF in bacterial genomes. To assess the dosage constraint, we overexpressed TF in either single TF-containing *E. coli* or multiple TF-containing *M. xanthus* strains. Subsequently, domain mutations of TF were conducted for similar overexpression experiments to determine the contributions of different domains. We assayed proteome changes in the TF overexpression *E. coli* mutant. We demonstrated the specific cellular functions of these N-terminal defect TF homologs in *M. xanthus*. Our work highlights that the intrinsic characteristics of TF, including ribosome binding and substrate-holding abilities, contribute to the dosage constraint of TF, which drives the evolution and fates of its duplicates in bacteria.

## RESULTS

### Almost all bacteria possess a single ribosome-binding TF

**Fig 1:**
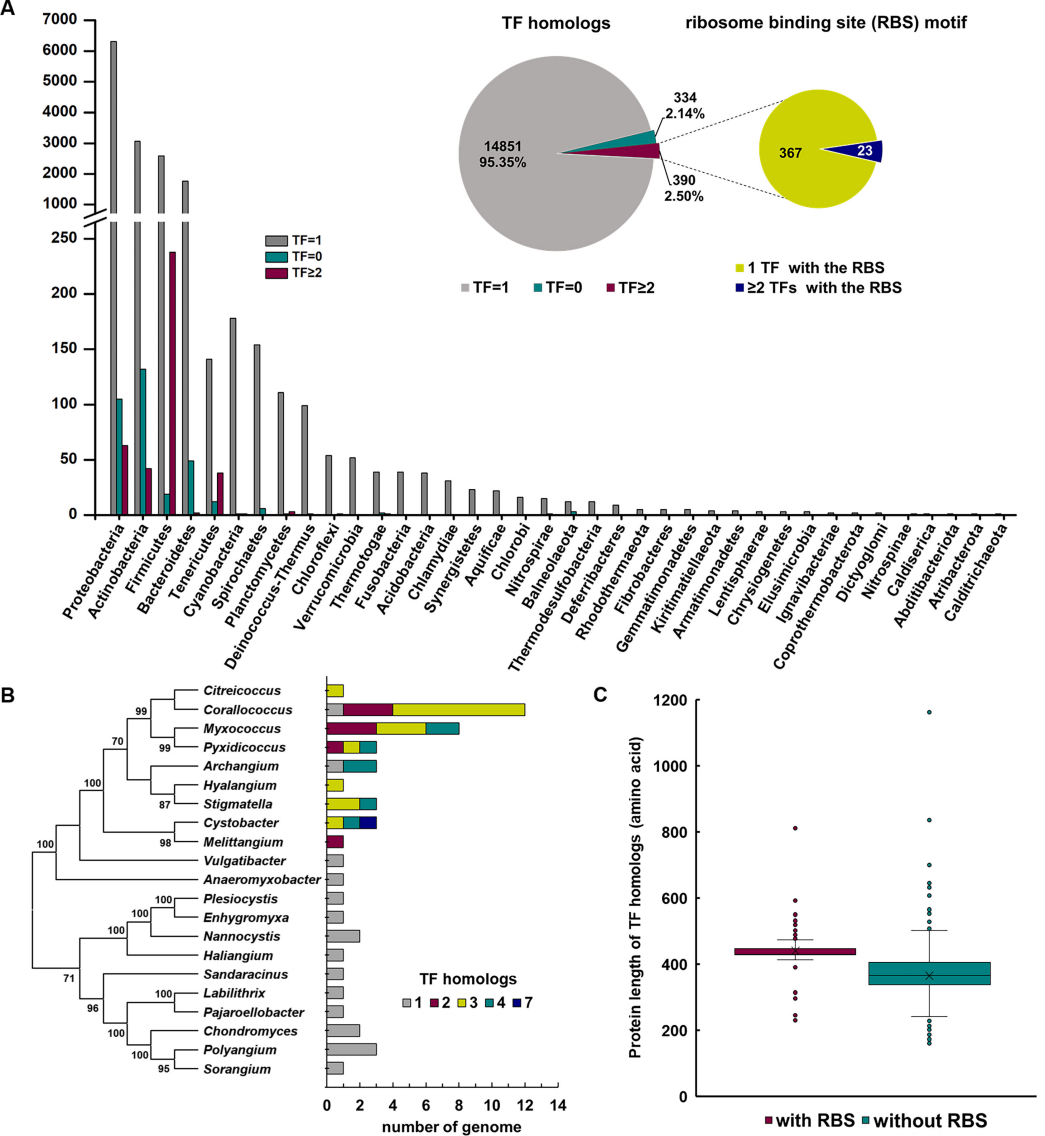
Analysis of the existence of TF homologs in representative bacterial genomes. (**A**) Number and ratio of genomes containing no, one, or multiple TF homologs, and the existence of the RBS motif in genomes with multiple TF homologs. (**B**) The number of genomes containing different copies of TF homologs in the genus *Myxococcales*. (**C**) Protein length analysis of TF homologs with or without RBS in 390 multiple TF-containing genomes. The crosses mark the average length of proteins, and the lines in boxes mark the median length of proteins.

We screened the existence of TF by searching for proteins belonging to the TIGR00115, COG0544, or PRK01490 CDD families (refer to Materials and Methods) in 15,575 representative bacterial genomes. In total, 97.86% (15,241) of these genomes contained at least one TF-encoding gene, and 334 were identified as lacking this gene (Data set S1). Among the TF-containing bacteria, 97.44% (14,851) had a single TF gene, while only 390 bacteria contained multiple copies of TF (Fig. 1A). Genomes with multiple TF homologs mainly occurred in highly sequenced phyla. Among the top five phyla containing multiple TFs, 209 out of the 238 *Firmicutes* genomes belonged to the class of *Clostridia*, and almost all the genomes with multiple TFs in *Tenericutes* belonged to an order of *Mollicutes*, *Mycoplasmatales* whose genomes are generally streamlined in small size (Table S1; for details, refer to Data set S1). These bacteria with multiple TF copies were widely distributed and not closely related to species, except for the *Myxococcales* cluster (Fig. S1A). In the *Myxococcales* order, there might be one, two, three, four, or even seven TF copies in the genome (Table S2), but multiple TF copies were only found in the *Cystobacterineae* suborder (Fig. 1B). The above results indicated that, different from the duplication frequency of other chaperones, e.g., 41.3% for DnaK (37) or 19.5% for GroEL (38), almost all bacteria possessed a single copy of TF, and the very few exceptions with multiple TF homologs were widely distributed across different taxonomic groups.

The conserved RBS motif of TF has been previously characterized by the GFR×G××P sequence composition (11). However, we found possible variations in RBS motifs such as GYR×G××P in some *Mycoplasmataceae* genomes or GFRKGK×× in some *Clostridia* and other genomes (Data set S1). We used the degenerate G×R×G motif to determine the presence of RBS in the N-terminal 100-amino acid sequences of TF proteins in bacteria harboring multiple TF homologs. Among the 390 genomes examined, 367 retained only one TF with the RBS motif, while the other TF homologs lacked this conserved motif (Fig. 1A; Data set S1). We found that the protein length of the TF homologs without RBS was significantly shorter than that of the RBS-containing TFs (Fig. 1C and *t*-test, *P*-value < 0.001; for details, refer to Data set S1). The reduced length was mostly due to the deficiency in the N-terminal domain, as shown by the protein sequence alignment of TF homologs in 33 myxobacteria with multiple TFs (Fig. S2 and S3).

The 23 bacteria with multiple RBS-containing TFs were phylogenetically dispersed (Fig. S1A). These TF homologs might exhibit low sequence identities and varied RBS sequences, such as the four RBS-containing TF homologs in *Sporomusa silvacetica* DSM 10669, or were highly similar, such as the TF homologs in *Filifactor alocis* ATCC 35896, *Salibacterium salarium*, *Dyella soli*, and *Petrotoga halophila* DSM 16923 (Table S3). In the completely sequenced genome of *F. alocis* ATCC 35896, there was a ~20,000 bp *tig*-containing repeat sequence, indicating that a small-scale duplication event occurred recently. Interestingly, the endogenous plasmid in *Mycoplasmopsis pulmonis* NCTC10139 contained an RBS-containing TF gene that was almost identical to that in the chromosome, whereas the congeneric *M. pulmonis* UAB CTIP with no plasmid contained only one TF gene in its chromosome. These findings suggested that multiple TF genes might have originated from either duplication or horizontal gene transfer, and would consequentially evolve into a single conserved TF chaperone with additional TF homologs that lacked the RBS-containing N-terminal domain. The existence analysis of TF in bacteria suggested that a dosage constraint was placed on the copy number of TF.

### *In vivo* dosage constraint of TF chaperone

**Fig 2:**
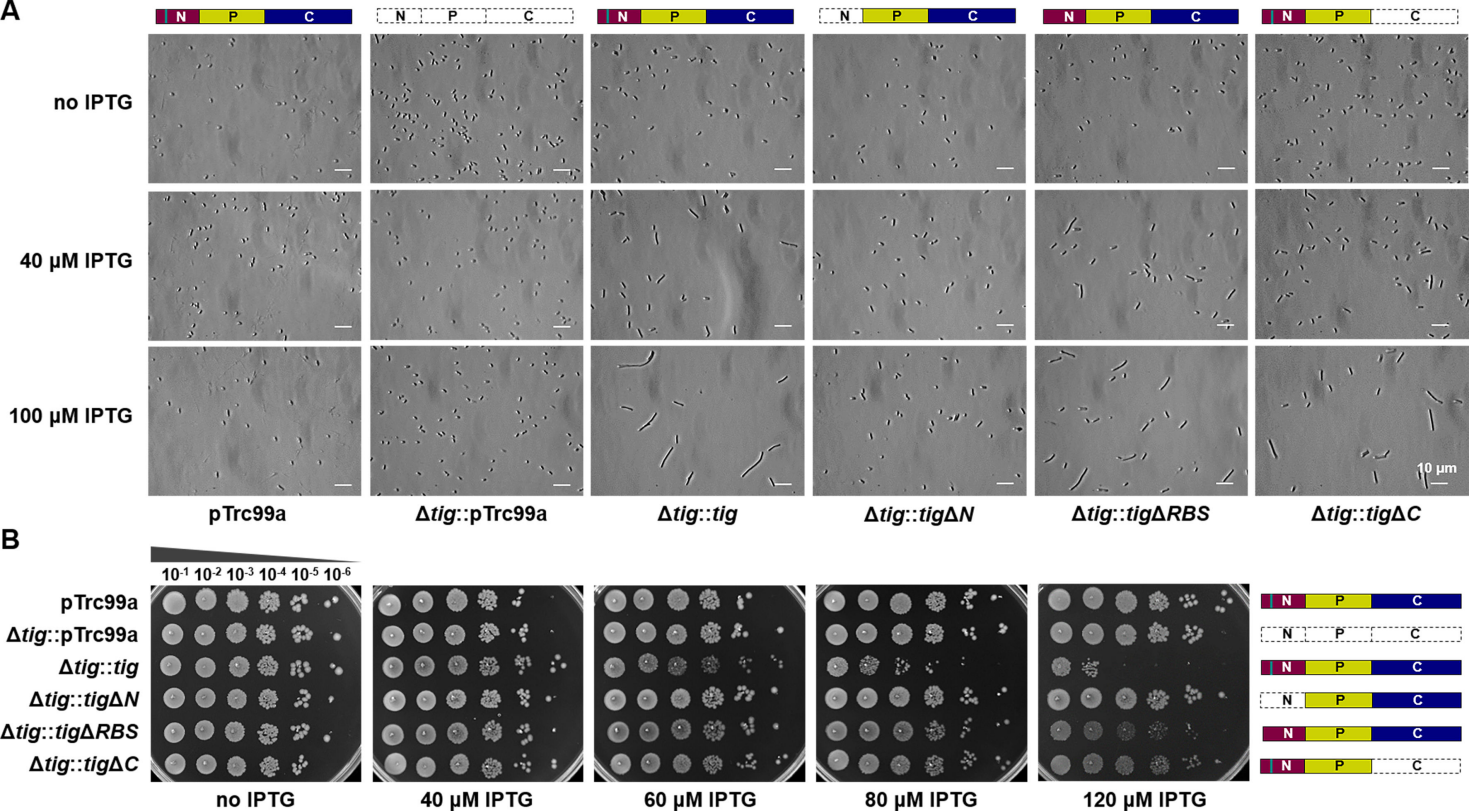
Physiological analysis of the various *tig* overexpressing *E. coli* mutants. (**A**) Cell filamentation of the mutants in liquid media without, 40 µM, or 100 µM IPTG. The cells were observed by a phase contrast microscope. The white bar represents 10 µm. (**B**) Growth of *E. coli* recombinant mutants induced by different concentrations of IPTG. Then, 1:10 serial dilutions (horizontal dimension) were spotted on Luria-Bertani (LB) plates containing 100 µg/mL ampicillin (Amp) with different concentrations of IPTG.

To investigate the possible dosage effects of TF proteins, we deleted the original *tig* gene from *E. coli* MG1655 and complemented *tig* with an isopropyl-beta-D-thiogalactopyranoside (IPTG)-inducible promoter in the pTrc99a plasmid (Δ*tig::tig*). The overproduction of TF proteins was observed in the Δ*tig::tig* mutant upon IPTG induction compared to the control strains pTrc99a (MG1655 with empty pTrc99a) and Δ*tig*::pTrc99a (*tig* deletion with empty pTrc99a) (Fig. S4A; the production was increased by more than 10 times when 80 µM IPTG induced, as shown by the proteome analysis below). Consistent with a previous report (29), the Δ*tig::tig* mutant cells were elongated with the addition of IPTG, and the filamentation increased at a high IPTG concentration of 100 µM (Fig. 2A). We observed that the filamentous cells were polyploid (Fig. S4B), suggesting that chromosome replication and division occurred in these abnormally divided cells. Additionally, the growth of the *tig*-complemented strain became weaker with increasing IPTG concentration and was strongly inhibited by 120 µM IPTG in the plates (Fig. 2B). The results indicated that excessive TF proteins were severely detrimental to the growth of bacterial cells.

We further constructed the overexpression mutant strains by replacing the complete TF with variant TFs, including the N-terminal domain-deleted TF (Δ*tig::tig*Δ*N*), the RBS motif-deleted TF (Δ*tig::tig*Δ*RBS*), and the C-terminal domain-deleted TF (Δ*tig::tig*Δ*C*), which were similarly induced with IPTG (Fig. S4A). The *tig*Δ*N* complemented mutant completely rescued the growth inhibition and cell filamentation caused by excessive TF (Fig. 2A and B). Notably, while the Δ*tig::tig*Δ*RBS* or Δ*tig::tig*Δ*C* mutant partially restored the inhibited growth, the cells still exhibited filamentation under IPTG induction. This result suggested that both ribosome binding and substrate-holding activities are involved in the growth inhibition caused by excessive TF, given that the RBS-containing N-terminal domain is implicated in ribosome binding and substrate holding (11, 12), and the C-terminal domain serves as the main substrate-holding region (8, 13, 14).

### N-terminal differences determine the dosage constraint of TF homologs

**Fig 3:**
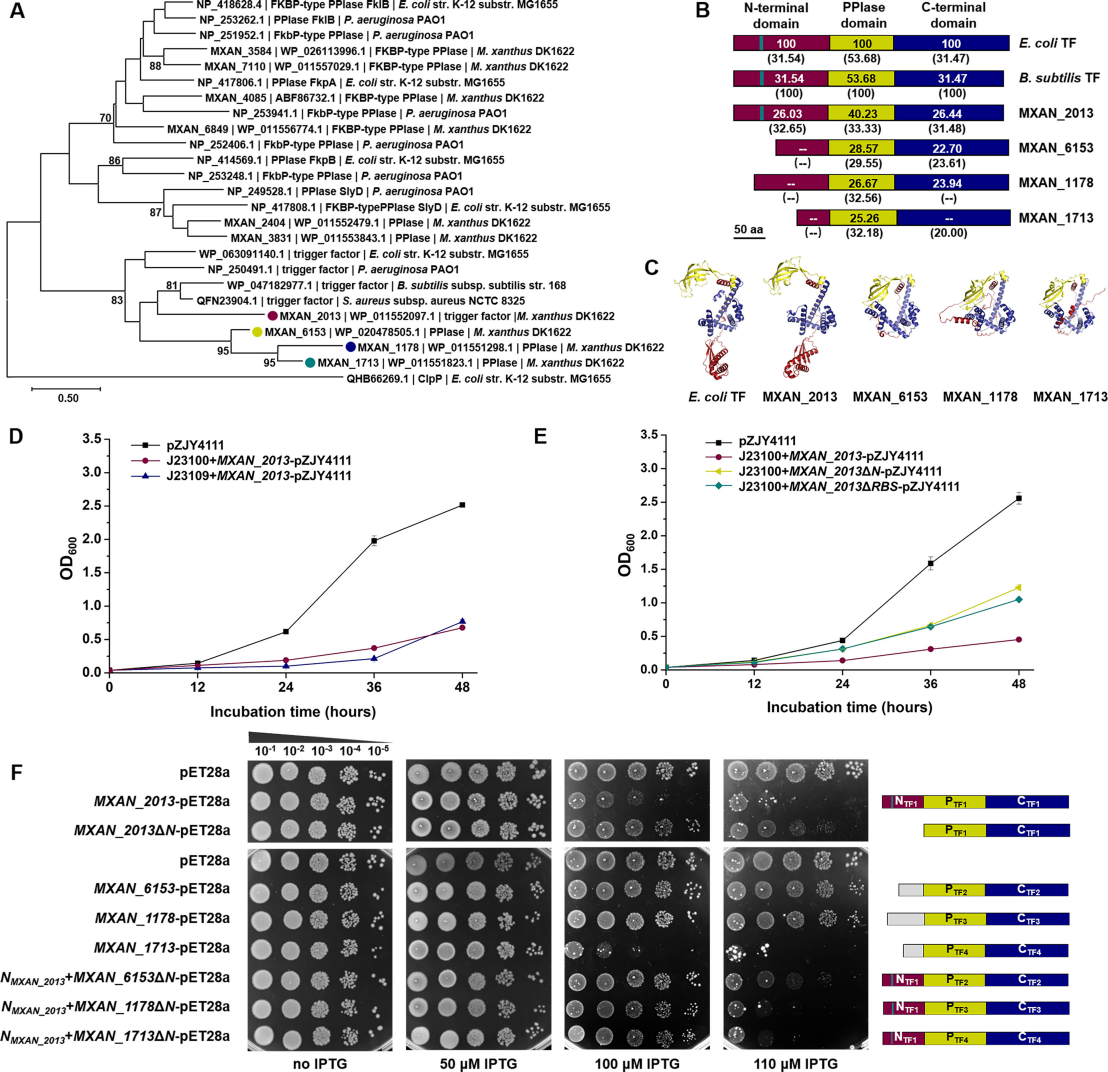
Growth inhibition assays of the four *M. xanthus* TF homologs. (**A**) Phylogenetic tree of FKBP-type PPIases in *M. xanthus* DK1622, *E. coli*, *B. subtilis*, *S. aureus*, and *P. aeruginosa*. Support values larger than 70% are presented. (**B**) Diagrammatic analysis and identity percentages of different domains of TF homologs. The numbers in or under the diagrams indicate the identity of this domain relative to *E. coli* TF or *B. subtilis* TF, respectively. The green bar marks the RBS motif and “--” means no identity. (**C**) Protein modeling analysis of MXAN_2013, MXAN_6153, MXAN_1178, and MXAN_1713 by AlphaFold 2. *E. coli* TF was used as the reference. The N-terminal, PPIase, or C-terminal domain is drawn in red, yellow, or blue, respectively. (**D**) Growth of *MXAN_2013*-overexpressing *M. xanthus* strains. J23109 and J23100 are the chosen promoters. (**E**) Growth of the *M. xanthus* mutants overexpressing *MXAN_2013*, *MXAN_2013*Δ*N*, and *MXAN_2013*Δ*RBS* using the J23100 promoter. (**F**) Growth analysis of heterologously expressed mutants for TF homologs in *M. xanthus* DK1622. *E. coli* BL21 was chosen as the heterologous host. All the LB plates contained 40 µg/mL Km to maintain the plasmids. The right image is a domain diagram of heterologously expressed TF homologs and fusion proteins.

We investigated the dosage constraint of the N-terminal complete TF proteins in multiple TF-containing bacteria. According to the genome annotation, the model strain of myxobacteria, *M. xanthus* DK1622, possesses 10 FKBP-type PPIases, including four TF homologs. Phylogenetic analysis of the protein sequences revealed that MXAN_2013 was grouped with the identified canonical TFs (39) to form the TF branch, while the other three TF homologs (MXAN_6153, MXAN_1178, and MXAN_1713) formed a separate neighboring branch (Fig. 3A). Together, these two branches constituted the clade of TF homologs. In comparison, other *M. xanthus* FKBP-type PPIases were mixed with non-TF FKBP-type PPIases from *E. coli*. Sequence comparison demonstrated that the three domains of MXAN_2013 are close to those of the TFs of *E. coli* or *B. subtilis*, while the other three *M. xanthus* TF homologs are in similar identities of the PPIase domain and the C-terminal domain but have different or no N-terminal domains (Fig. 3B). Specifically, MXAN_2013 possesses a conserved RBS motif of GFR×G××P, whereas the other three homologs lose this motif entirely. Protein modeling analysis with the AlphaFold2 program indicated that the PPIase domain and the C-terminal domain of the four *M. xanthus* TF proteins are similar, but only MXAN_2013 has an N-terminal extension similar to that of the *E. coli* TF (Fig. 3C).

We employed several classical strategies to overexpress these TF homologs in *M. xanthus* DK1622 but were unsuccessful. Subsequently, we selected 10 promoters with different strengths that are workable in *M. xanthus* (40) and individually constructed the promoters in the autonomous replication plasmid pZJY4111 (41, 42) to regulate the expression of *MXAN_2013*. Among these promoters, only J23109 and J23100 exhibited functionality by upregulating the transcription of *MXAN_2013* by 2.6- and 3.3-fold, respectively (Fig. S5A). Nevertheless, this modest increase in transcription significantly inhibited the growth of *Myxococcus* strains (Fig. 3D). Similarly, we constructed overexpression mutants of *MXAN_2013*Δ*N* and *MXAN_2013*Δ*RBS* using the J23100 promoter, which resulted in an approximately twofold increase in transcription (Fig. S5A). The growth of the two mutants was partially restored compared to that of the *MXAN_2013* overexpression mutant (Fig. 3E). However, the phenomenon of filamentization was not observed in these *M. xanthus* overexpression mutants (Fig. S5B).

Although TF was not highly conserved in different species (Fig. 3B), the overexpression of *tig* from *B. subtilis* could also inhibit the growth of *E. coli* (Fig. S5C), possibly due to the conserved RBS and spatial structure of TF. We heterologously expressed each of the four *M. xanthus* TF homologs in *E. coli* BL21. We also constructed *E. coli* recombinant strains with the N-terminal deleted TF (*MXAN_2013*Δ*N*) and TF chimeras by replacing the N-terminal domain with that of *MXAN_2013* in three other TF homologs, namely *N_MXAN_2013_-MXAN_6153*Δ*N*, *N_MXAN_2013_-MXAN_1178*Δ*N*, and *N_MXAN_2013_-MXAN_1713*Δ*N* (Fig. S5D). All the recombinant strains grew normally without IPTG induction (Fig. 3F). After the 110 µM IPTG induction, the *E. coli* strains with *MXAN_6153* or *MXAN_1178* grew normally, but those with *N_MXAN_2013_-MXAN_6153*Δ*N* or *N_MXAN_2013_-MXAN_1178*Δ*N* were significantly inhibited. The growth of the recombinant strain with *MXAN_1713* was abnormal due to the inclusion body form of MXAN_1713 proteins in *E. coli* cells (Fig. S5E). When the N-terminal domain of *MXAN_1713* was replaced, the growth of the recombinant strain with *N_MXAN_2013_-MXAN_1713*Δ*N* was rescued under 100 µM IPTG induction but still severely inhibited at 110 µM IPTG, similar to the growth of strains with *N_MXAN_2013_-MXAN_6153*Δ*N* or *N_MXAN_2013_-MXAN_1178*Δ*N*. The results in *M. xanthus* DK1622 indicated that the dosage constraint appeared in the N-terminal complete TF but not in the N-terminal defect TF homologs.

### Excessive TF decreases protein synthesis and stabilizes TF-substrate complexes

**Fig 4:**
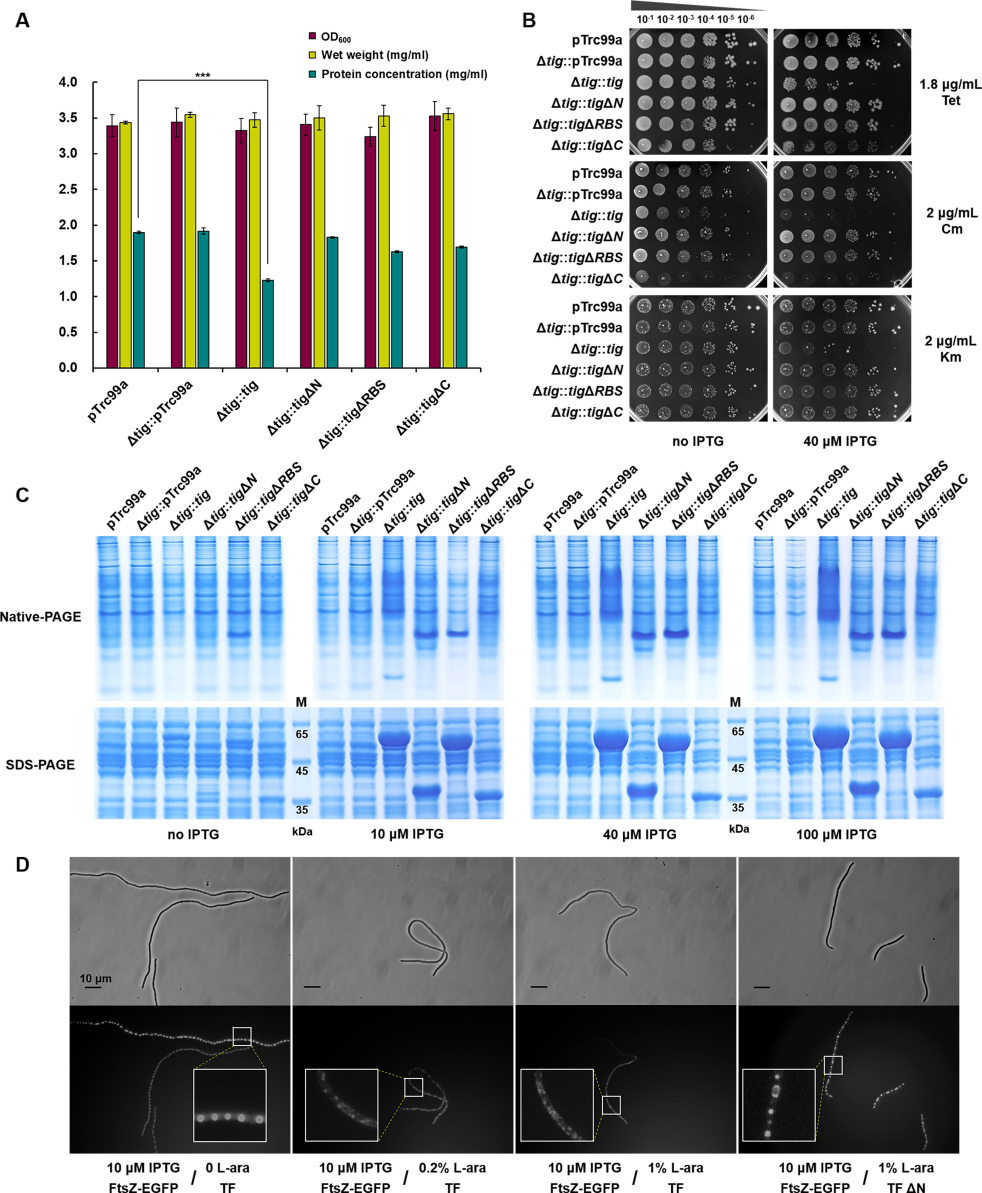
Changes in protein production in different *E. coli* overexpression mutants. (**A**) The OD_600_, wet weight, and protein concentration of *E. coli* mutants after incubation for 6 h with 100 µM IPTG. For statistical analysis, ***, *t*-test, *P*-value < 0.001. All the LB plates and liquids contained 100 µg/mL Amp to maintain the plasmids. (**B**) Growth of *E. coli* mutants under 1.8 µg/mL tetracycline (Tet, which inhibits polypeptide synthesis by acting on the 30S subunit of the ribosome), 2 µg/mL chloramphenicol (Cm, which prevents mRNA binding to the ribosome by acting on the 50S subunit), or 2 µg/mL kanamycin (Km, which interferes with protein synthesis by binding the 16S rRNA of the 30S subunit of the ribosome) with 0 or 40 µM IPTG. (**C**) Native-PAGE and SDS-PAGE of whole-cell proteins of *E. coli* mutants after induction with different amounts of IPTG. The loaded protein was the soluble component after ultrasonic breaking and centrifugation. (**D**) Z-ring formation analysis under different induced concentrations of TF and TFΔN proteins. The locations of the Z-rings were observed by FtsZ-EGFP using fluorescence phase contrast microscopy (lower panel). The black bar represents 10 µm.

Since controlling the expression of TF homologs in *M. xanthus* is challenging, we used *E. coli* to investigate the factors leading to the dosage constraints of TF and the subsequent consequences. After induction with 100 μM IPTG, the protein concentration of the Δ*tig::tig* mutant significantly decreased to 64.7% of the pTrc99a strain, while the protein concentration of the mutant strains Δ*tig*::pTrc99a and Δ*tig::tig*Δ*N* were similar to that of the pTrc99a strain; these strains exhibited no significant differences in growth (Fig. 4A). The decrease in total protein concentration indicated that overproduced TF impacted protein biosynthesis, but there was no reduction in the relative protein level of ribosomal proteins (Fig. S6A). We chose three ribosome-binding antibiotics (tetracycline, chloramphenicol, and kanamycin), and two ribosome non-binding antibiotics (phosphonomycin and polymyxin) to check the influences of TF overproduction by assaying the antibiotic sensitivity changes in different *tig* mutants. When induced by 40 µM IPTG, the growth of Δ*tig::tig* was significantly inhibited after small amounts of tetracycline (acting on the 30S subunit), chloramphenicol (acting on the 50S subunit) or kanamycin (binding to 16S rRNA) were added. In addition, the growth of Δ*tig::tig*Δ*C* was inhibited by the addition of tetracycline or chloramphenicol but not kanamycin, whereas the growth of Δ*tig::tig*Δ*N* and Δ*tig::tig*Δ*RBS* was not inhibited under the same conditions (Fig. 4B). With respect to the ribosome non-binding antibiotics phosphonomycin and polymyxin, there was no significant difference in the growth of these recombinant mutants after IPTG induction (Fig. S6B and C). The enhanced growth inhibition caused by ribosome-binding antibiotics in conjunction with redundant RBS-containing TF proteins further suggested that the consequence of excessive TF was related to the protein synthesis function of ribosomes.

Among the TF overexpression mutants, only the complete *tig* overexpression strain showed obvious dispersive bands on native polyacrylamide gel electrophoresis (native-PAGE), and these bands became more pronounced with increasing IPTG induction (Fig. 4C). These dispersive bands could be pulled down with TF-His by Ni-NTA in the TF-His overexpressed *E. coli* BL21 strain (Fig. S6D). The results suggested that redundant TF in cells over-captured the substrate proteins, which was consistent with previous *in vitro* experiments showing that TF proteins over-captured predenatured GAPDH or bovine carbonic anhydrase II at a high concentration (35, 43), leading to the maintenance of substrates in the unfolded state in intermediate complexes with TF (15). Consequently, redundant TF proteins likely contributed to the formation of stable intermediate complexes of TF and substrates, causing them to maintain an unfolded state *in vivo*.

Overexpression of TF led to abnormal cell filamentation, therefore, we fused the self-assembling cell division protein FtsZ (44, 45) with the enhanced green fluorescent protein (EGFP, fluorescent label) to show the polymerization of the Z-ring in the TF-overproduced *E. coli* mutant. The protein production induced by different concentrations of inducers was shown in Fig. S6E. FtsZ-EGFP induced by 10 µM IPTG was observed to form stable light spots via fluorescence microscopy, revealing the location of the Z-ring (Fig. 4D). However, overexpression of TF, but not TFΔN, resulted in diffuse fluorescence, suggesting that excessive amounts of TF inhibited the formation of Z-ring. These results were consistent with the filamentization of *tig*-overexpressed *E. coli* cells and the recovery of abnormal filamentization in the *tig*Δ*N* overexpression strain. Overall, redundant TF proteins affected the process of protein synthesis in ribosomes, probably due to their heavy binding to ribosomes and over-capturing substrates, forming stable intermediate complexes of some functional proteins.

### Overproduction of TF leads to proteome profile changes

**Fig 5:**
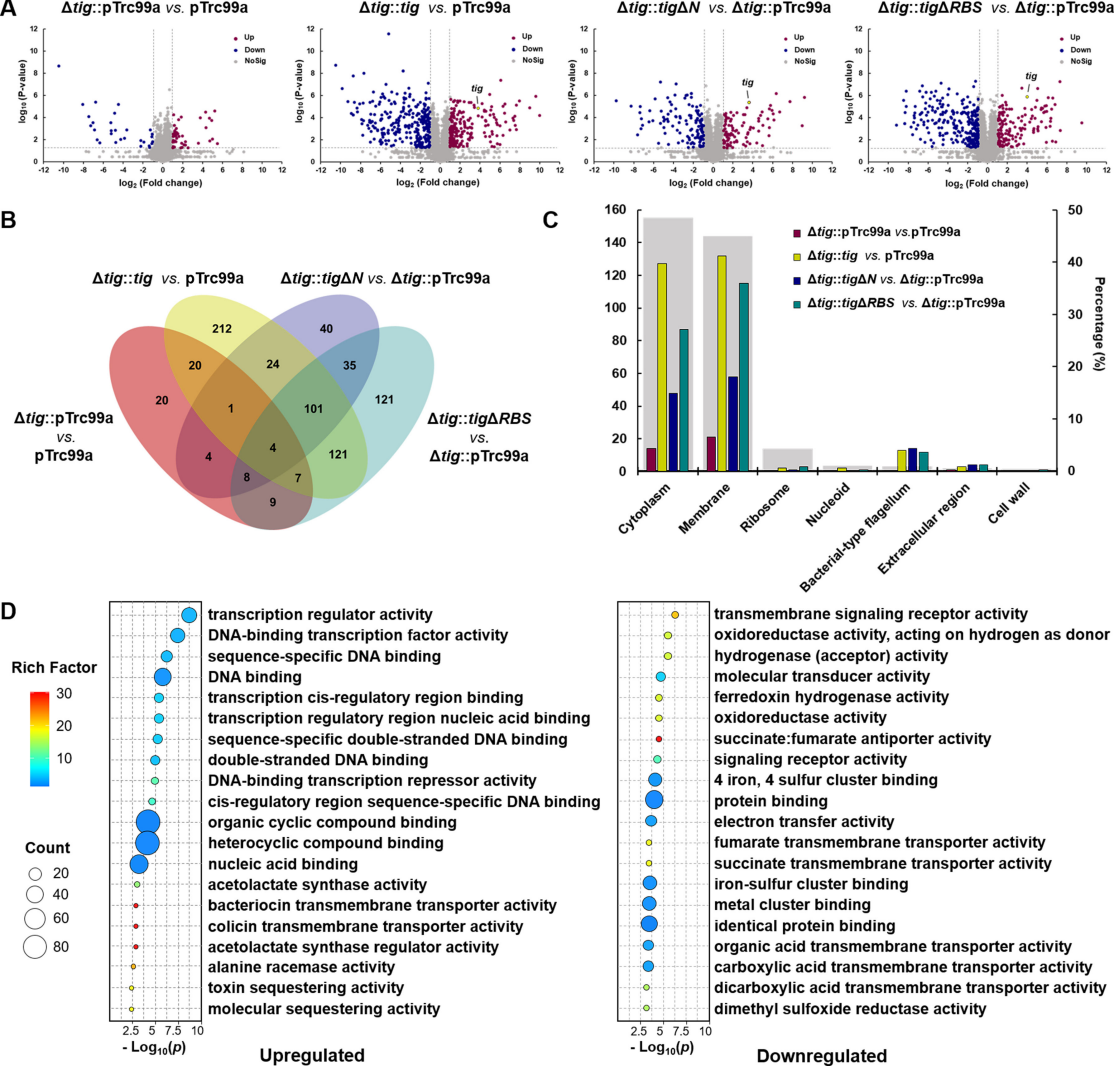
Proteomic analysis of the various *tig* overexpressing *E. coli* mutants. (**A**) Volcano plots of DEPs. (**B**) Venn diagram of the number of DEPs. (**C**) The counts of DEPs at different subcellular locations. The gray columns indicate the percentage (right y-axis) of proteins distributed in the corresponding component compared with total proteins. The thresholds for differential expression were selected by fold change >2 and *P*-value < 0.05 (*t*-test). (**D**) Top 20 molecular functions enriched from DEPs in the *tig* overexpressing strain compared with the wild-type strain with empty pTrc99a.

We conducted 4D label-free quantitative proteomics analysis on different mutant strains after the induction with 80 µM IPTG. Compared with the wild-type strain with empty pTrc99a (pTrc99a), the knockout of *tig* (Δ*tig*::pTrc99a) caused slight proteome differences (Fig. 5A). This finding was consistent with previous results in which the deletion of TF had no impact on growth. However, the overexpression of *tig* (Δ*tig::tig*) led to significant changes in proteome composition, which were partially alleviated by deleting the N-terminal domain (Δ*tig::tig*Δ*N*) (Fig. 5A and B). The protein levels of TF or its variants in Δ*tig::tig*, Δ*tig::tig*Δ*N*, and Δ*tig::tig*Δ*RBS* were approximately 14-fold, 13-fold, and 16-fold greater than the original TF level in the wild-type strain with pTrc99a, respectively (for details, see Data set S2). Differential expression proteins (DEPs) in the *tig* overexpression mutant appeared to be enriched in cell membrane components (Fig. 5C; similar results were found in the Gene Ontology knowledgebase analysis, referred to Fig. S7A), consistent with previous results showing that TF is involved in the biosynthesis of membrane proteins (9, 46, 47). However, the DEPs in the *tig*Δ*N* overexpression mutant were significantly reduced in almost all the protein categories (Fig. S7A). These results suggested that TF overexpression had a global effect on the proteome profile, but this effect could be reduced by deleting the N-terminal domain.

In total, 204 upregulated DEPs and 286 downregulated DEPs were observed when *tig* was overexpressed (fold change >2, *P*-value < 0.05; for details, see Data set S2). The top-upregulated DEPs were mostly related to nucleic acid binding, while the top-downregulated DEPs were associated with oxidoreductase activity and metal ion binding ability (Fig. 5D), possibly because the hydrophobic cavity of TF tends to bind to alkaline amino acids with positive charge, aromatic residues, and hydrophobic residues (14, 48). Among the 181 TF-interacting proteins in *E. coli* identified by Martinez-Hackert (49), 54 were upregulated and 33 were downregulated in the *tig* overexpression mutant (*P*-value < 0.05, Table S4).

The overexpression of TF resulted in abnormal cell division and dispersed Z-ring, whereas the level of cell division-associated proteins increased in the *tig* overexpression mutant (Fig. S7B). For example, the FtsZ protein was upregulated when TF was overexpressed. Similarly, the DNA translocase and divisome FtsK, which is recruited to the divisome soon after FtsZ (50) and has been determined to interact with TF to regulate cell division in *Staphylococcus aureus* (51), also showed upregulation when *tig* was overexpressed. Moreover, the protein level of ZapC, which stabilizes the polymeric form of the cell division protein FtsZ (52), increased by approximately 60-fold in the *tig* overexpression mutant. These results suggested that these TF interacting proteins may be overcaptured by redundant TF proteins and thus inhibited their maturation; therefore, they were upregulated to complement their cellular functions when TF was overproduced. Overall, redundant TF proteins caused protein profile changes, which can be greatly restored by the deletion of the N-terminal domain. These results further confirmed that the N-terminal domain played a key role in the impact caused by the overproduction of TF.

### N-terminal defective TF homologs have evolved divergent functions

**Fig 6:**
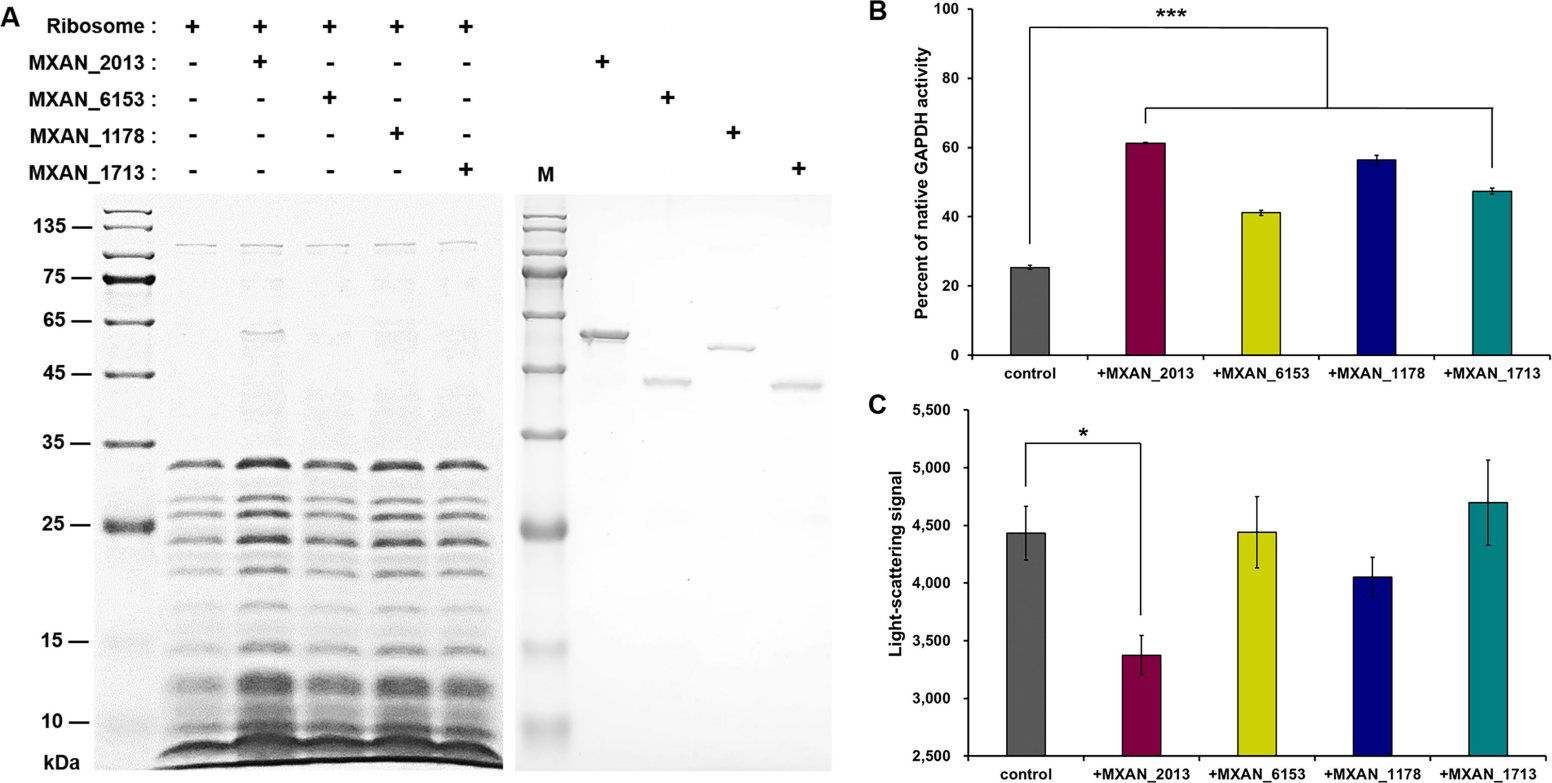
*In vitro* functional analysis of TF homologs in *M. xanthus* DK1622. (**A**) Binding detection of *M. xanthus* DK1622 ribosomes with MXAN_1178, MXAN_1713, MXAN_2013, or MXAN_6153. The 4 µM purified protein was incubated with ribosomes separately and subjected to sucrose cushion centrifugation. The sediments were resuspended and subjected to SDS-PAGE (left). The right figure shows the SDS-PAGE of the purified proteins of *M. xanthus* TF homologs. (**B**) Refolding activities of TF homologs. The recovered activity of predenatured GAPDH was measured by the yield of NADH in 60 s, which was detected by the absorbance at 450 nm. The percentage was calculated relative to that of the natural enzyme. (**C**) Prevention of the aggregation of predenatured GAPDH by TF homologs. Denatured GAPDH was 100-fold diluted in buffer with 1 µM TF proteins, followed by an increase in the light-scattering signal at 620 nm. For statistical analysis, *, *t*-test, *P*-value < 0.05; ***, *t*-test, *P*-value < 0.001.

We also detected the characteristics and cellular functions of these defect TF homologs to investigate the reason for their retention during evolution. The four *M. xanthus* TFs were heterologously expressed in *E. coli* BL21, purified, and subsequently incubated with ribosomes of *M. xanthus* DK1622 (Fig. S8A). Only MXAN_2013, but not the other three TF homologs, co-sedimented with ribosomes after centrifugation on the sucrose cushion (Fig. 6A), which was consistent with the sequence analysis (Fig. 3B). By using GAPDH as the substrate, we confirmed that these TF proteins all exhibited refolding capabilities. Compared to the spontaneous 25.4% recovery without any addition of TF protein, all four TF homologs significantly increased the recovery to 41.2%–61.2% (Fig. 6B; *t*-test, *P*-value < 0.001). In terms of preventing aggregation of predenatured GAPDH proteins, only the addition of MXAN_2013 but not the other three proteins significantly decreased the light scattering signal (Fig. 6C; *t*-test, *P*-value < 0.05). Thus, although the N-terminal RBS-containing domain played a critical role in the classical function of TF, the extra TF homologs had lost this domain, especially the RBS motif, possibly to avoid the dosage constraint of this ribosome-associated molecular chaperone.

**Fig 7:**
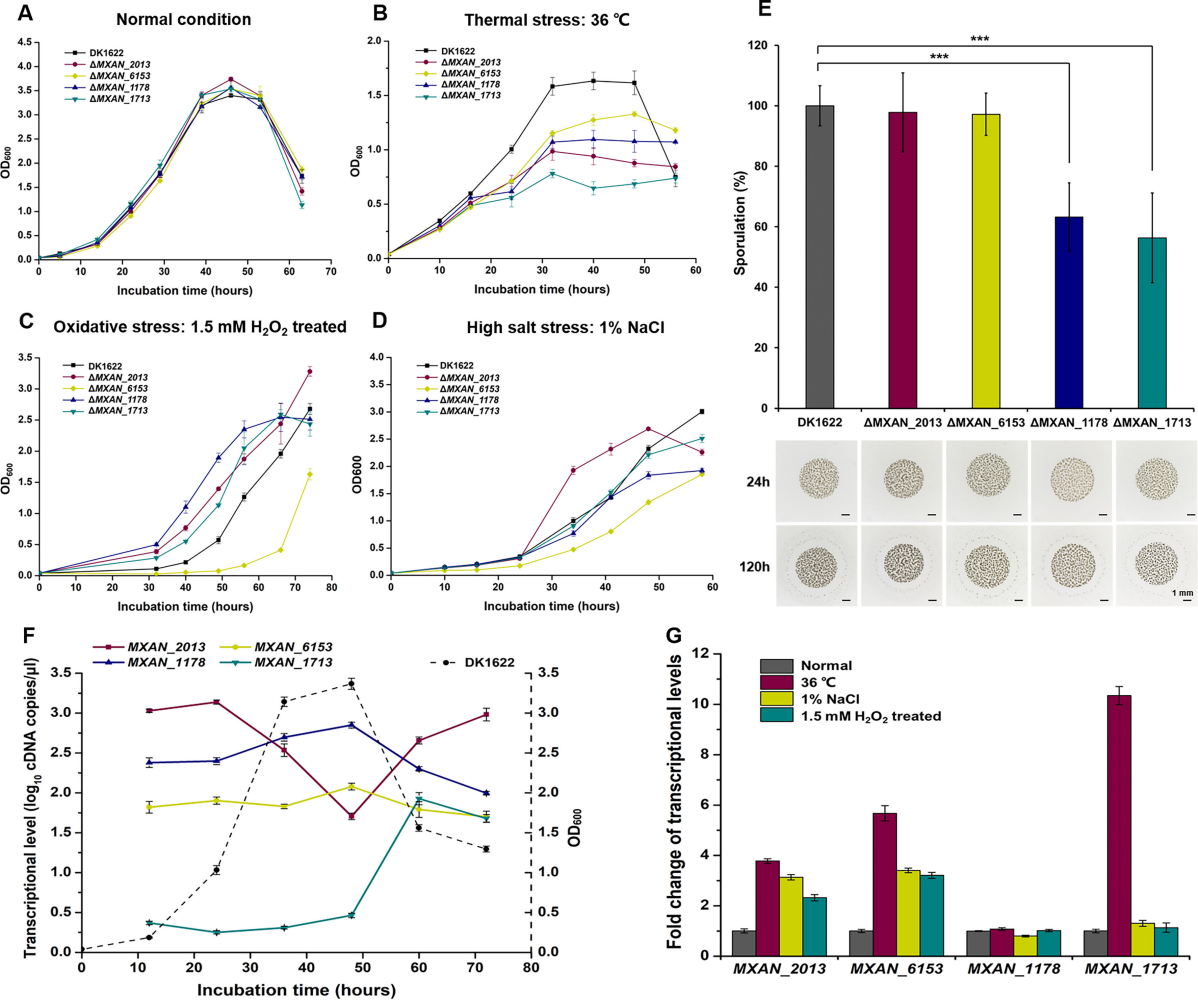
Cellular physiological functions and gene transcriptional levels of four TF homologs in *M. xanthus* DK1622. Growth curves of the *MXAN_1178*, *MXAN_1713*, *MXAN_2013*, and *MXAN_6153* knockout mutants, and the wild-type strain DK1622 under normal conditions at 30°C (**A**), high temperature at 36°C (**B**), after treatment with 1.5 mM hydrogen peroxide (H_2_O_2_) for 30 min (**C**), and with 1% NaCl added (**D**). Cell densities were measured with an ultraviolet spectrophotometer at 600 nm. (**E**) Sporulation ability of *M. xanthus* mutants after starvation-induced on TPM plates for 120 h; the percentages relative to those of DK1622 were calculated. For statistical analysis, ***, *t*-test, *P*-value < 0.001. The bottom pictures show fruiting body development induced by starvation on TPM plates after 24 h and 120 h. The black bar represents 1 mm. (**F**) Transcription levels of *MXAN_2013*, *MXAN_6453*, *MXAN_1178*, and *MXAN_1713* at different time points during incubation. The concentration of cDNA after RNA reverse transcription was measured. The log_10_ values of cDNA concentration are shown. *gapA* was used as the reference gene. (**G**) Quantitative PCR (qPCR) analysis of the fold changes in transcriptional levels of *MXAN_2013*, *MXAN_6153*, *MXAN_1178*, and *MXAN_1713* in *M. xanthus* DK1622 under different stress conditions. The transcriptional level of each gene under normal conditions was set to 1. *gapA* was also used as a reference gene.

We separately knocked out the four TF homologs in *M. xanthus* DK1622, and all the deletion mutants exhibited a similar growth curve to that of the wild-type strain at the normal growth temperature of 30°C (Fig. 7A). When the incubation temperature was increased to 36°C, the growth of *M. xanthus* DK1622 cells was impacted, while the four knockout mutants grew more weakly, with the *MXAN_1713* deletion mutant displaying the most compromised growth (Fig. 7B). Similarly, the growth abilities of the wild-type strain DK1622 and the knockout mutants were significantly inhibited by the H_2_O_2_ treatment for 30 min (Fig. 7C) or the 1% NaCl addition (Fig. 7D), and the *MXAN_6153* deletion mutant exhibited the weakest growth under these conditions.

Myxobacteria are characterized by their unique and complex social behavior (53, 54). We compared the social characteristics of the knockout mutants and the wild-type strain. The A- and S-motilities of the mutants were similar to those of the DK1622 strain and different from that of the A-motility-deficient strain MxH2265 (Δ*aglZ*, A^−^) (55), or the S-motility-deficient strain DK10410 (Δ*pilA*, S^−^) (56) (Fig. S8B). Similarly, there were no significant differences in predation or morphogenesis of fruiting bodies between the mutants and the wild-type strain. However, the sporulation abilities of the *MXAN_1178* and *MXAN_1713* knockout mutants decreased to 63.2% and 56.4% of that of the wild-type strain, respectively (Fig. 7E; *t*-test, *P*-value < 0.001).

We observed significant variations in the transcription levels of *MXAN_2013*, *MXAN_6153*, *MXAN_1178,* and *MXAN_1713* throughout the growth curve (Fig. 7F). In the early exponential growth stage, *MXAN_2013* exhibited the highest transcription level, whereas *MXAN_1713* showed extremely low transcription level but significantly increased in the decline phase. In comparison, the transcription levels of *MXAN_1178* and *MXAN_6153* were moderate and slightly changed during the incubation time. The transcriptional change trend of *MXAN_2013* in different growth periods was similar to that of *groEL1* in *M. xanthus* DK1622 (57), suggesting potential cooperative functions between them. We noticed that the transcriptional changes of these TF homologs were consistent with their divergent functions. For example, the transcription of *MXAN_2013* was upregulated in response to thermal, oxidative, or salinity stresses, whereas *MXAN_1713* was significantly upregulated only at thermal conditions (Fig. 7G). *MXAN_6153* was also upregulated under thermal, oxidative, or salinity stress conditions, which aligned well with the phenotypes observed in the *MXAN_6153* deletion mutant. Although the transcription of *MXAN_1178* did not exhibit any noticeable response to any of the tested stress conditions, a previous study demonstrated a twofold upregulation in the transcription of *MXAN_1178* during sporulation (58). This finding supported our result that *MXAN_1178* was associated with the sporulation process of myxobacteria.

We performed pull-down and mass spectrometry to identify the proteins that potentially interact with the four TF homologs. The results revealed some partial overlaps among their potential substrates, but most were unique (Fig. S8C; Table S5). Interestingly, certain proteins that were identified as classical substrates of TF, such as some ribosomal proteins (49) and OmpA (46), were absent in the possible substrates of MXAN_2013 but present in those of the other three homologs. It suggested that these four TF homologs performed divergent functions, which was consistent with our phenotypic findings. Overall, the results suggested that after losing the ribosome-binding ability and being deficient in the N-terminal, the extra TF homologs avoiding the dosage constraint are retained, and undergo evolutionary divergence to participate in TF functions by subfunctionalization.

## DISCUSSION

Gene duplication is an important evolutionary process that provides new genetic material for organisms to acquire new functions (59). However, the most duplicated genes are silenced in evolution, and the few survivors, which are generally ABC-type transporters, transcription factors, and dehydrogenases in bacteria (60), subsequently undergo strong purifying selection. It is unclear how duplicate genes are lost or successfully retained from functional redundancy. As the only ribosome-associated molecular chaperone in bacteria, TF plays a vital role in refolding and stabilizing cellular proteins and is present in almost all bacterial species. Nevertheless, very few bacteria (2.5%) possess multiple TF homologs; notably, the majority of them retain one complete TF while others are mutated in the N-terminal region. Herein, we report that although TF is pivotal for the biosynthesis of proteins and maintenance of homeostasis, the presence of TF is subjected to a quantitative limitation, which accounts for the loss of duplicated *tig* genes in most bacteria.

**Fig 8:**
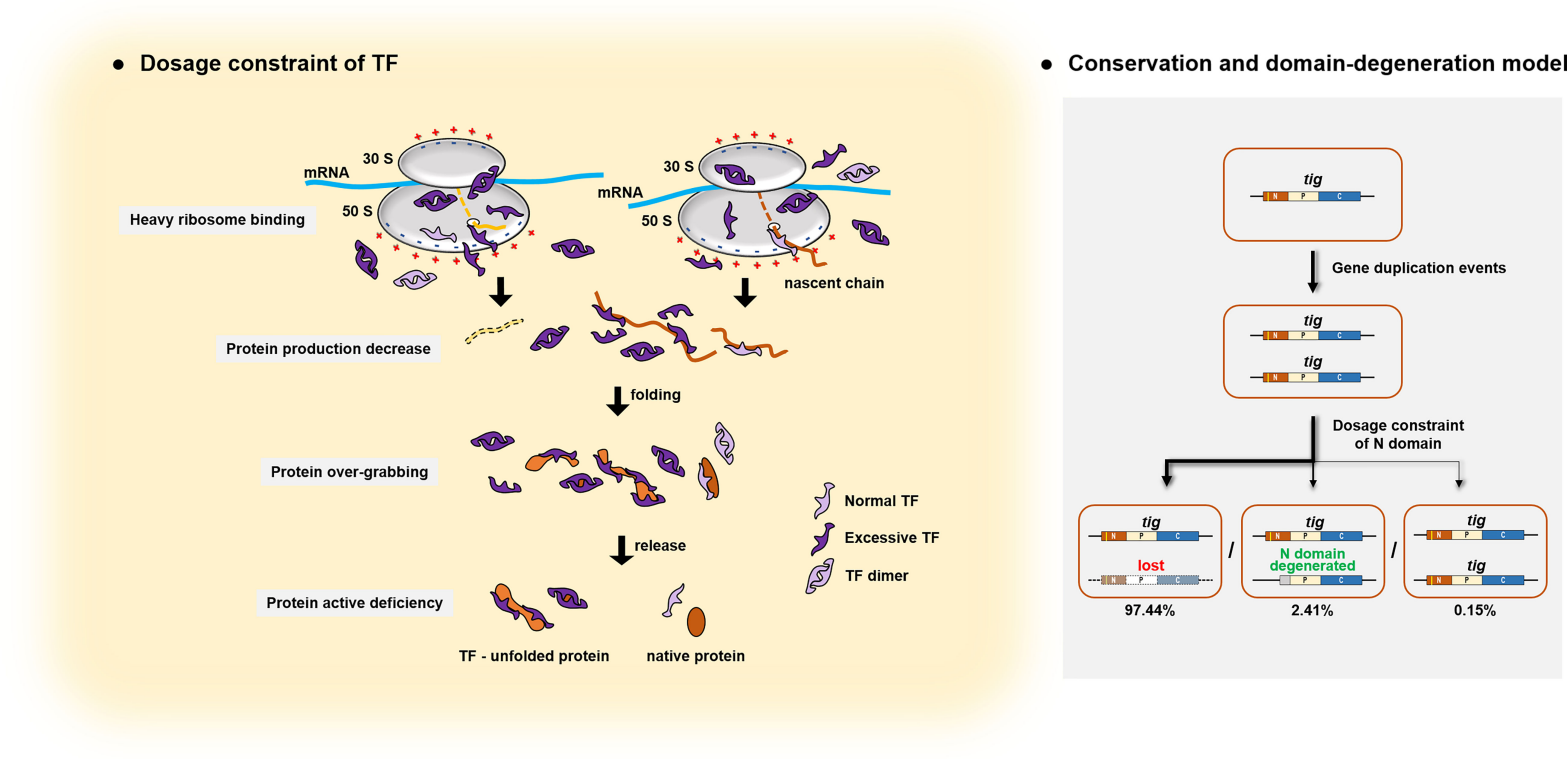
Diagrammatic model for the dosage constraint and evolutionary fates of TF duplicates. The characteristic cellular function of TF provides the structural basis for its dosage constraint. We suggested that redundant TF heavily occupies the binding site at the tunnel exit rim of the nascent peptide chain on the ribosome, which impedes the synthesis of peptides and causes a decrease in protein production. Outside ribosomes, redundant TF proteins overcapture substrates in their immature unfold state and inhibit their complete release from TF proteins *in vivo*, which results in a deficiency of active proteins even if the amount of protein is available. Gene duplication events presumably occurred in *tig*; however, due to the dosage constraint of TF, the extra *tig* gene was rapidly lost during evolution. Meanwhile, the disorder caused by redundant TF proteins could be completely reversed by removing the RBS-containing N-terminal domain. Thus, the extra *tig* copy with the degenerated N domain could avoid the dosage constraint and evolve along with the conserved one for retention. As a result, except for the TF-absent genomes, 97.44% of the TF-containing representative bacterial genomes possess a single TF gene, and 2.41% retained one complete copy while the rest copies are defective at the N-terminal and possibly evolve into divergent functions, only the rest 0.15% exist more than one complete TF copy.

Among the three functional domains of TF, the N-terminal domain contains an RBS motif responsible for its capacity to bind ribosomes (11). Upon binding to ribosomes, TF provides the first hydrophobic binding platform for synthesized polypeptide chains (14, 61). Assisted by the TF chaperone, substrates are efficiently folded into their native state and subsequently released from TF; for those unfolded substrates, TF holds them to prevent their autonomous polymerization (15). The TF protein can be present in excess of the ribosome *in vivo* (62), although TF-ribosome binding occurs at a 1:1 stoichiometry (63). However, the overexpression of TF is detrimental to bacterial cells, and the detrimental effects can be completely rescued by deleting the N-terminal domain of TF, or partially recovered by removing the RBS motif or C-terminal region. Considering that the C-terminal domain is responsible for substrate binding, while the N-terminal domain is associated with ribosome binding and substrate-holding activities (12, 14, 64, 65), the damage caused by TF overproduction may result from the over-holding of substrate proteins inhibiting the release of substrates, as well as excessive ribosome binding impeding the synthesis of peptides. Although the impact of overexpression cannot fully represent the effects brought about by gene duplication, our work demonstrates the two-sidedness of TF functions and establishes a correlation between the intrinsic characteristics of TF and the evolutionary consequences of its duplicated genes. The diagrammatic model depicted in Fig. 8 elucidates the possible mechanism that underlies the dosage constraint of TF.

We previously demonstrated in *M. xanthus* DK1622 that the duplicates of GroELs (57, 66–68) and DnaKs (37, 69) are functionally divergent. The four TF homologs in *M. xanthus* DK1622 also display differential effects on stress tolerance and social behavior. In bacteria, TF is involved in various cellular processes based on the functions of substrate proteins, including large complex assembly, ribosome biogenesis, outer membrane protein biosynthesis, protein secretion, virulence, stress tolerance, competence development, and biofilm formation (9, 26, 47, 70–72). In *M. xanthus*, the deletion of the RBS-containing *MXAN_2013* did not significantly impact growth, social behavior, or resistance to stress, except for thermal stress. Among the other three TF homologs that are deficient in the N-terminal region, MXAN_6153 plays a significant role in resistance to oxidative stress and hypersaline stress, which are typical functions of TF reported in other bacteria (25, 70). In contrast, both *MXAN_1178* and *MXAN_1713* are involved in the myxobacterial sporulation process, and *MXAN_1713* also participates in the response to thermal stress. Consistently, the substrates of these TF homologs are varied. Three alternative outcomes may occur during the evolution of duplicate genes: nonfunctionalization to loss, neofunctionalization that expands to new beneficial functions, or subfunctionalization that shares the total capacity of the ancestral gene (4). Our findings demonstrate that the N-terminal defect TF homologs not only remain partial cellular functions of the ribosome-associated TF chaperone but also expand some new specific cellular functions such as social behavior. Thus, these N-terminal deficient TF homologs in *M. xanthus* partition the function of TF by subfunctionalization.

Whether a duplicate gene is retained depends upon its function, its mode of duplication, the species in which it occurs, and its expression rate (73). When gene duplication of *tig* occurs, a conserved copy is always retained while the extra copies would be silenced through mutation and lost due to the dosage constraint. The N-terminal domain degenerated copies might be preserved and undergo further functional selection, resulting in the subfunctionalization of these duplicates, which, however, lose the ribosome binding capacity and related cellular functions. Our findings suggest that, in addition to the accumulation of mutations as the evolutionary force, an internal limitation factor occurs within the protein itself: sites or domains associated with dosage constraint are prone to loss or mutation shortly after gene duplication events.

The asymmetric evolution of gene duplications is underappreciated, partly because the origin of highly divergent genes can be difficult to resolve (74). “Escape from adaptive conflict (EAC)” has been proposed as a mechanism of subfunctionalization of gene duplications, in which one copy is selected to improve ancestral function while the others are free to improve novel function (75). Generally, each of the duplicated genes optimizes function divergently from the multi-functions possessed by the ancestral gene (5). The evolutionary fate of TF homologs conforms to the EAC model but with some differences. The evolution process of duplicate genes of TF is highly uneven; one is almost unchanged to maintain the TF fundamental functions as the ribosome-associated molecular chaperone, while the others lose the N-terminal domain that is for the ribosome binding capacity and related functions. We propose the following “conservation and domain-degeneration model” (Fig. 8) to describe this special evolution pattern of gene duplication: due to the domain dosage constraint and functional necessity of protein, one conserved copy with complete structure and basic function always exists, while the extra copies will lose during evolution, or degenerate the deterministic domain to avoid the dosage constraint and are retained for functional expansion.

## MATERIALS AND METHODS

### Bioinformatics analysis of the TF homologs in prokaryotic genomes

The conserved domain of TF was obtained from the CDD protein family TIGR00115 (trigger factor), COG0544 [FKBP-type peptidyl-prolyl cis-trans isomerase (trigger factor)], and PRK01490 (trigger factor; provisional). The proteins containing these conserved domains were, respectively, identified by the TIGRFAMs, COGs, and PRK protein databases for the annotation of TF, and all these annotations were integrated into the NCBI genome database (76). To identify the homologous proteins of TF in prokaryotes, we searched for proteins that best matched the TIGR00115, COG0544, or PRK01490 CDD families in 15,575 representative bacterial genomes. We excluded proteins that were less than 120 amino acids in length because the conserved PPIase domain of TF is approximately 120 amino acids in length. The RBS was screened at the beginning of 100 amino acids of the N-terminal domain of TF proteins.

The phylogenetic analysis was performed using MEGA 11. Sequence alignment was performed using the online program MAFFT (https://www.ebi.ac.uk/Tools/msa/mafft/). Sequence consistency was analyzed by NCBI protein BLAST (https://blast.ncbi.nlm.nih.gov/Blast.cgi). The spatial structure of the proteins was modeled by AlphaFold 2.

### Strains and growth conditions

The strains and plasmids used in this study are listed in Table S6. The *E. coli* strains were routinely grown in Luria-Bertani (LB) medium (Tryptone 1%, Yeast extract 0.5%, NaCl 1%). The solid media were created by adding 1.5% agar. *M. xanthus* strains were cultivated in casitone-based rich-nutrient (CTT) media [1% casitone, 8 mM MgSO_4_, 10 mM Tris-HCl (pH 7.6), 1 mM K_2_HPO_4_-KH_2_PO_4_; pH 7.6] (77). To induce the development and spore formation of *M. xanthus* strains under starvation conditions, TPM agar media [10 mM Tris-HCl (pH 7.6), 8 mM MgSO_4_, 1 mM K_2_HPO_4_-KH_2_PO_4_, and 1.5% agar] (78) were used. For mutant selection, ampicillin (Amp) was added to the medium at the final concentration of 100 µg/mL, and kanamycin (Km) was added at a final concentration of 40 µg/mL. *M. xanthus* was grown at 30°C, and *E. coli* was grown at 37°C unless otherwise noted.

### Construction of plasmids and strains

The *tig* gene in *E. coli* MG1655 was deleted using a method described previously (79). The complete *tig* gene, *tig*Δ*N* (with the N-terminal domain deleted), *tig*Δ*C* (with the C-terminal domain deleted), and *tig*Δ*RBS* (with the RBS deleted) were cloned and inserted into the IPTG-induced plasmid pTrc99a using the ClonExpress II One Step Cloning Kit (Vazyme) to construct the recombinant expression plasmids, which were subsequently transformed into the *tig*-deleted *E. coli* strains separately.

The *ftsZ-egfp* gene was cloned and inserted into pTrc99a. We used the L-arabinose-induced plasmid with the *tig* gene to separately induce the TF and FtsZ-EGFP proteins. These two plasmids were both transformed into *E. coli* MG1655.

Overexpression of *MXAN_2013* and its variants in *M. xanthus* DK1622 was achieved by the autonomous replication plasmid of *M. xanthus*, pZJY4111 (41, 42). The promoters of J23109 or J23100, the ribosome binding site (BBa_B0034), and *MXAN_2013* or its mutant genes were cloned and inserted into pZJY4111 and subsequently transferred into DK1622 cells via electrotransformation.

Gene deletion in *M. xanthus* DK1622 was performed using positive-negative KG cassettes (80). Upstream and downstream homologous fragments of the genes, each 800–1,000 bp, were amplified by Phanta Super-Fidelity DNA Polymerase (Vazyme) and cloned into pBJ113 to construct the deletion plasmids. The deletion plasmid was electroporated into DK1622 cells, and individual Km-resistant colonies were chosen. Then the positive transformants were diluted and spread onto CTT agar plates supplemented with 1.5% D-galactose (Sigma) for a second selection. The deletion mutant candidates were confirmed by PCR and sequencing.

The primers used for PCR amplification are listed in Table S7.

### *E. coli* growth test

The growth of the recombinant *E. coli* mutants was tested by culturing them at 37°C in LB medium until they reached a final concentration of OD_600_ = 1.0. After 10-fold serial dilution, 3 µL of each dilution was dropped onto LB agar containing the required concentration of antibiotics and different concentrations of IPTG.

The morphologies of *E. coli* mutant cells cultured in liquid media supplemented with different concentrations of IPTG were observed under a phase contrast microscope.

### Total protein concentration detection

*E. coli* recombinant strains were collected after culture at 37°C for 6 h. The total protein of cells was extracted by using a Bacterial Protein Extraction Kit (Sangon). The protein concentration was determined using a modified BCA Protein Assay Kit (Sangon).

### Native-PAGE assay

The cells of the *E. coli* mutants were collected after incubation in a liquid medium for 6 h with different IPTG concentrations at 37°C and then resuspended and crushed by ultrasonication. The cell-broken mixtures were centrifuged at 13,000 rpm for 15 min at 4°C to obtain soluble proteins in the supernatant. The protein concentration was adjusted to 30 mg/mL, and the protein was mixed with a native loading buffer. The native-PAGE system was precooled on ice, and the experiment was carried out at 4°C. Tris-Glycine native-PAGE running buffer (pH 8.8) was purchased from Sangon.

### 4D label-free quantitative proteomics

The analysis of 4D label-free quantitative proteomics (81) was performed by Shanghai Bioprofile Technology Co., Ltd. The collected cells were homogenized using liquid nitrogen, and total protein was extracted by the addition of the SDT lysate. The samples were boiled and further ultrasonicated. Undissolved cellular debris was removed by centrifugation at 16,000 × *g* for 15 min. Protein quantification was carried out using a BCA Protein Assay Kit (Sangon), with each experimental group consisting of three biological replicates. Proteins were digested and collected by the filter-aided sample preparation method, and the peptides were desalted using a C18 reversed-phase column prior to freeze-drying. Then, the peptides were redissolved in 0.1% trifluoroacetic acid for liquid chromatography-mass spectrometry analysis and separated using an nLC 1200 chromatography system. MS data acquisition was performed using a timsTOF Pro2 mass spectrometer operated in PASEF mode.

MS data were analyzed with MSFragger 3.4 software, and proteins were identified from the UniProt Protein Data Bank (Swissprot-*Escherichia coli* [562]-23228-20221128.fasta). The identified proteins were annotated using the Gene Ontology knowledgebase, and GO enrichment analysis was performed using Fisher’s exact test.

### Purification of His-tag proteins

Each gene was cloned and inserted into pET28a using a ClonExpress II One Step Cloning Kit (Vazyme) to construct the protein expression vectors. These vectors were subsequently transformed into *E. coli* BL21 for heterologous expression. The His-tagged proteins were purified as previously described (33). The purified proteins were assayed for concentration using an ultramicrospectrophotometer and detected by SDS-PAGE. M lane represents the protein marker (M5 HiClear Prestained Protein Ladder MF212, Mei5 Biotechnology Co., Ltd.).

### Ribosome purification and binding test

Previous methods were used to purify the ribosomal proteins from *M. xanthus* DK1622 and verify the binding between TF homologs and ribosomal proteins (65). Ribosomal proteins were detected by SDS-PAGE. Equal amounts of ribosomal proteins were incubated with 4 µM TF homologous proteins at 25°C for 1 h. The reaction mixture and ribosome buffer supplemented with 20% sucrose solution were added to a centrifuge tube at a volume ratio of 1:3. The mixtures were centrifuged at 213,000 × *g* for 1 h, after which the pellets were resuspended. After the resuspension and recentrifugation at 12,000 rpm for 15 min, the supernatants of different samples were detected by SDS-PAGE.

### Prevention of aggregation and refolding assay

We measured the chaperone activity of the four TF proteins in *M. xanthus* DK1622 using previous methods (12, 20, 36) with some modifications. GAPDH (Sigma; G-2267) was used as the substrate for the chaperone activity tests. 125 µM GAPDH was denatured by incubation in 3 M GdnHCl with 5 mM DTT at 4°C overnight. To determine the effects of inhibiting protein aggregation, denatured enzymes were diluted 100-fold in GAPDH buffer (0.1 M potassium phosphate, pH 7.5, 1 mM EDTA, 5 mM dithiothreitol) containing 1 µM of each TF homologous protein. The buffer without TF protein was used as a control. The 90° light scattering signal at 620 nm was detected with a spectrofluorometer (Thermo Fisher; F-4600) after incubation for 10 min at room temperature.

The denatured protein refolding experiment was carried out by diluting the denatured GAPDH 100-fold into a GAPDH buffer containing 1 µM of each TF homologous protein. The reaction mixtures were incubated at 4°C for 30 min and then incubated at room temperature for 120 min. A GAPDH Enzyme Activity Assay Kit (Sigma; Mak277-1KT) was used to measure the enzyme activity of the reaction mixtures by measuring the absorbance at 450 nm with a spectrophotometer. GAPDH converts NAD^+^ to NADH, which has characteristic absorption at 450 nm. The accumulation of NADH in 60 s was used to determine the enzyme activities in various reaction systems. A reaction with an equal amount of natural GAPDH served as a positive control, while a reaction without TF protein served as a negative control. The activity percentage of all the experimental groups was calculated relative to that of the positive control.

### Growth and resistance analysis of *M. xanthus* strains

The growth and stress tolerance of *M. xanthus* strains were tested according to previous methods (37). Strains were inoculated in liquid CTT medium with 200 rpm shaking at 30°C for about 24 h (OD_600_ ≈ 1). The cultures were then inoculated into a CTT liquid medium at a final cell concentration of OD_600_ = 0.04 and incubated at 30°C with 200 rpm shaking. The cellular concentration was measured every 8 h.

After preliminary screening under different gradient conditions, we selected 36°C as the high-temperature pressure condition. For oxidative stress, the seed culture concentration was adjusted to OD_600_ = 1, and H_2_O_2_ was added to a final concentration of 1.5 mM. The cells were cultured for 30 min at 30°C with 200 rpm shaking before being inoculated into CTT liquid media. For the high-salt environment, 1% NaCl was added to the CTT liquid.

### Social behaviors of *M. xanthus* strains

The swarm assay was performed using a previously described method (54). Briefly, *M. xanthus* cells were harvested at the mid-log phase, washed three times with TPM buffer (10 mM Tris-HCl, 8 mM MgSO_4_, 1 mM K_2_HPO_4_-KH_2_PO_4_; pH 7.6), and adjusted to a final cell concentration of 5 × 10^9^ cells/mL. Then 2 µL aliquots were dropped onto 1.5% and 0.4% CTT agar media, and incubated at 30°C. The expansion of the swarm edges on 0.4% agar plates was observed with a stereoscopic microscope at regular intervals. The swarm edges on the 1.5% agar plates were observed with a phase contrast microscope after culturing for 72 h.

The predation assay was conducted according to a previous method (82). *M. xanthus* and *E. coli* cells were harvested at the mid-log phase and then washed three times with TPM buffer (pH 7.6). The *M. xanthus* cells were concentrated to a final density of 5 × 10^9^ cells/mL, and the concentration of *E. coli* was 1 × 10^11^ cells/mL. Then, 35 µL of *E. coli* cells were pipetted onto the TPM plate to form a cell mat with a diameter of approximately 1 cm, and 5 µL of *M. xanthus* cells were added to the center of the mat. The plates were incubated at 30°C, and the predation of *M. xanthus* on the *E. coli* mat was observed by stereoscopic microscopy.

For the sporulation assay, *M. xanthus* cells were harvested at the mid-log phase, washed, and resuspended in TPM buffer (pH 7.6) to a concentration of 5 × 10^9^ cells/mL. Then, 8 µL aliquots were placed onto TPM agar and incubated at 30°C. Fruiting body formation was observed with a stereomicroscope at different times. Sporulation was measured from the 5-day TPM cultures as previously described (57) and sporulation percentages were calculated as the number of colonies of deletion mutants divided by that of the wild-type strain DK1622.

### Quantitative PCR analysis

Quantitative PCR (qPCR) analysis was carried out as described previously (38). *M. xanthus* DK1622 cells were inoculated at a final concentration of OD_600_ = 0.04 in CTT media and cultured at 30°C with 200 rpm shaking. Subsequently, small portions were taken out from cultures for RNA extraction every 12 h. The qPCR detection of *M. xanthus* DK1622 under stress conditions or *M. xanthus* overexpression strains was sampled at OD_600_ = 1. The RNA was extracted using a Bacterial Total RNA Isolation Kit (Sangon). Genomic DNA removal and reverse transcription of RNA into cDNA were performed using HiScript IV RT SuperMix for qPCR (Vazyme). The cDNA was subsequently subjected to qPCR analysis using AceQTM Universal SYBR qPCR Master Mix (Vazyme). The *gapA* (a glyceraldehyde-3-phosphate dehydrogenase-encoding gene, *MXAN_2815*) gene was used as the reference gene for *M. xanthus* following a previous method (33). The linear curve of concentration versus cycle threshold (Ct) values was measured for each pair of primers, and Ct*_gapA_* = 25 was used to normalize the result obtained for each sample. The amplification efficiency of each pair of primers was as follows: E*_MXAN_2013_* = 102.72%, E*_MXAN_6153_* = 96.96%, E*_MXAN_1178_* = 96.25%, E*_MXAN_1713_* = 101.18%, and E*_gapA_* = 99.79%.

The primers used for qPCR analysis are listed in Table S7.

### Pull-down assay

The pull-down assay was performed according to a previously described method (38). We cloned the four TF homologous genes of *M. xanthus* DK1622 into the pMAL-c5x plasmid to produce proteins with the maltose binding protein (MBP) label. The purified MXAN_2013-MBP, MXAN_6153-MBP, MXAN_1178-MBP, and MXAN_1713-MBP proteins were incubated with the total protein of their corresponding deletion *M. xanthus* cells. Then, the mixture was passed through Dextrin Beads and washed with Tris-HCl buffer (25 mM Tris-HCl, 200 mM NaCl; pH 7.8). The MBP-labelled proteins could bind with Dextrin Beads, and their potential interacting proteins could be pulled down simultaneously. The Dextrin Beads binding proteins were washed out by MBP buffer (10 mM maltose, 25 mM Tris-HCl, 200 mM NaCl; pH 7.8) and detected by MS. The negative control was the total protein of DK1622 with MBP proteins.

## Data Availability

The data sets used and/or analyzed in the study are available in the article and the supplemental material.

## SUPPLEMENTAL MATERIAL

Data set S1 mbio.01994-24-s0001.xlsx The existence of TF homologs.

Data set S2 mbio.01994-24-s0002.xlsx Proteomic analysis.

Supplemental information mbio.01994-24-s0003.pdf Figures S1 to S8; captions for Tables S1 to S7 and Data sets S1 and S2.

Supplemental tables mbio.01994-24-s0004.xlsx Tables S1 to S7.

ASM does not own the copyrights to Supplemental Material that may be linked to, or accessed through, an article. The authors have granted ASM a non-exclusive, world-wide license to publish the Supplemental Material files. Please contact the corresponding author directly for reuse.
