# Supplementary material for: Dosage constraint of the ribosome-associated molecular chaperone drives the evolution and fates of its duplicates in bacteria
Source: mBio. 2024 Oct 7;15(11):e01994-24. doi: 10.1128/mbio.01994-24 (PMC11559001; doi:10.1128/mbio.01994-24)
Supplement: Supplemental information — Figures S1 to S8; captions for Tables S1 to S7 and Data sets S1 and S2. [file mbio.01994-24-s0003.pdf]

Supplementary information for

Dosage constraint of the ribosome-associated molecular chaperone drives the evolution and fates of its duplicates in bacteria

Tianyu Wan[1], Li Zhuo[1, 2, 3, *], Zhuo Pan[1], Rui-yun Chen[1], Han Ma[1], Ying Cao[1], Jianing Wang[1], Jing-jing Wang[1], Wei-feng Hu[1], Ya-jun Lai[1], Muhammad Hayat[1], Yue-zhong Li[1, *]

*The corresponding authors

Email: Yue-zhong Li: lilab@sdu.edu.cn

Li Zhuo: zhuoli1992@sdu.edu.cn

This PDF file includes:

Figures S1 to S8

Legends for Tables S1 to S7, Datasets S1, and Datasets S2

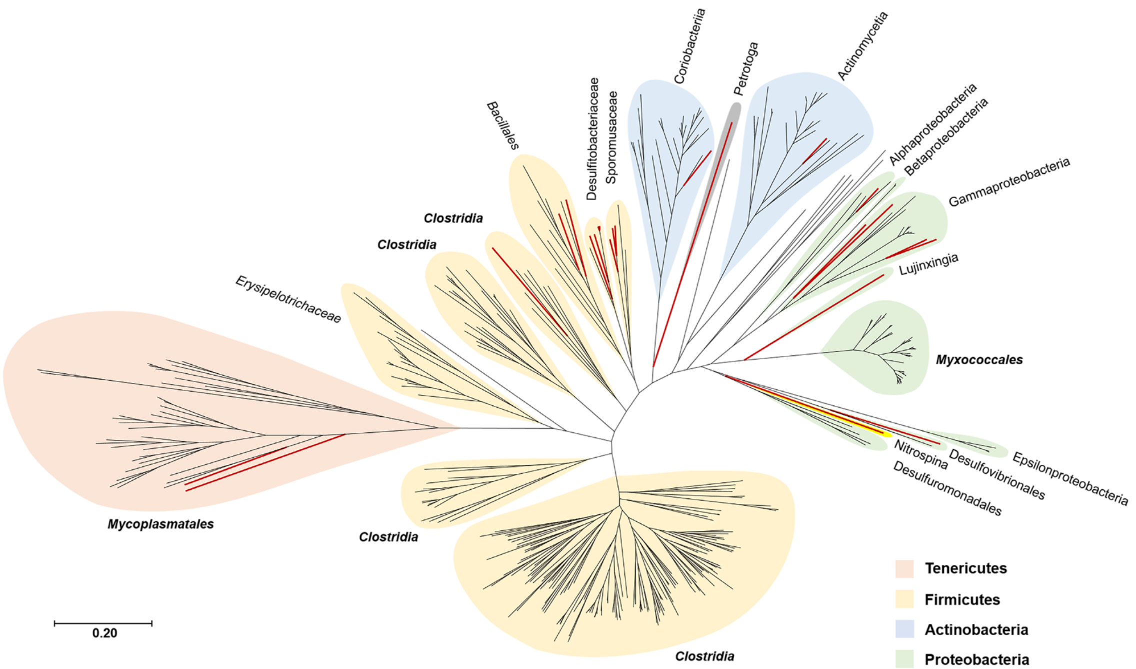

Fig. S1. Phylogenetic tree of 390 genomes with multiple TF homologs constructed by the whole genome sequence, related to Figure 1. The red branches show the genomes with multiple RBS-containing TF copies. The different colors distinguish different phyla.

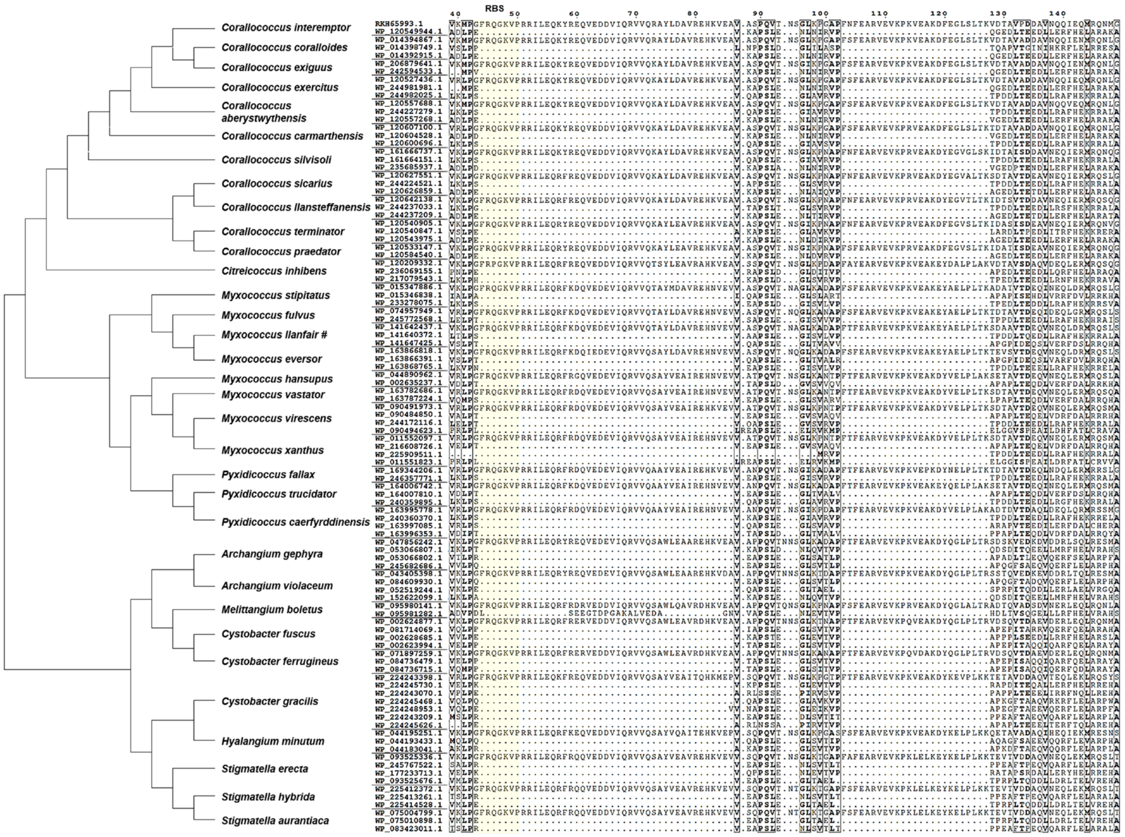

Fig. S2. Sequence alignment (partial presentation, the 40 to 150 aa) of TF homologs in 33 representative genomes of *Myxococcales* with multiple TF homologs, related to Figure 1. The RBS motif (GFR×G××P) is marked by the yellow background. The species name *Myxococcus llanfairpwllgwyngyllgogerychwyrndrobwllllantysiliogogogochensis* is abbreviated as *M. llanfair* and labeled with “#”. The length of the WP_224245626.1 protein is approximately 700 amino acids, and we selected only the TF homologous region.

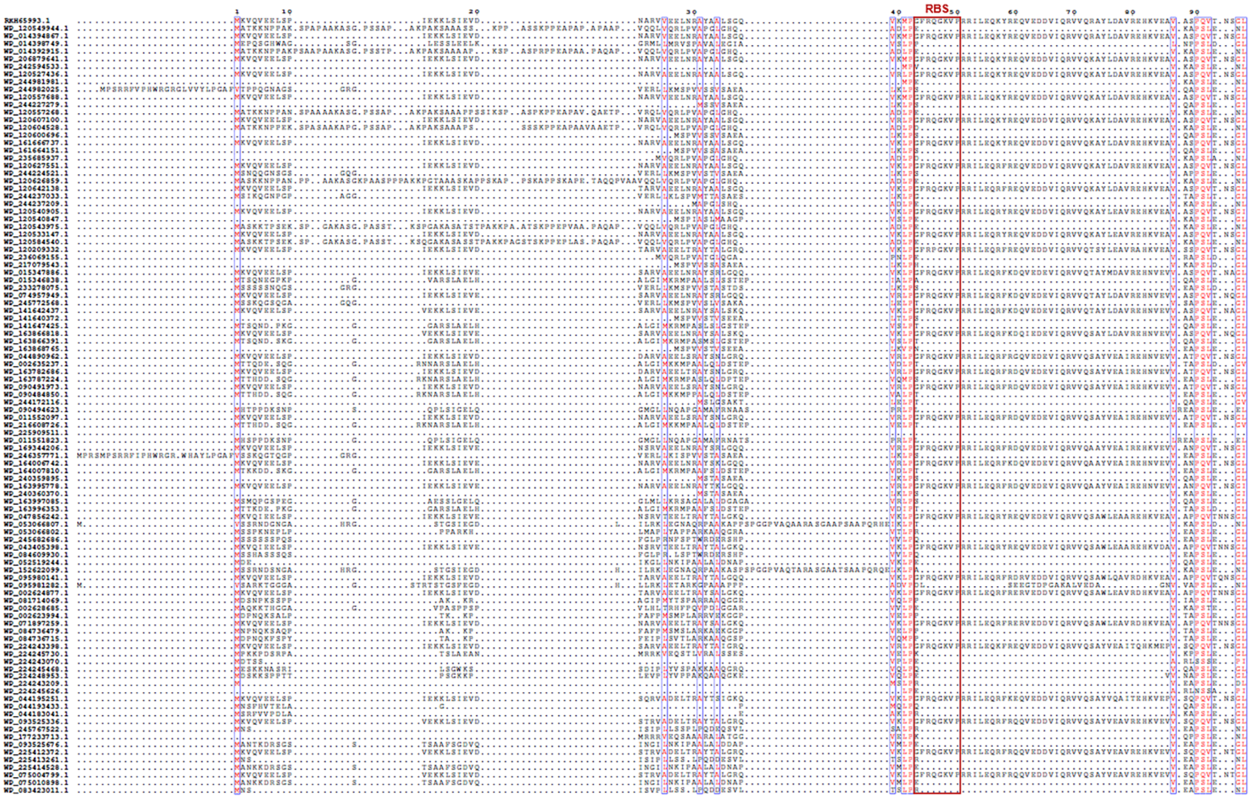

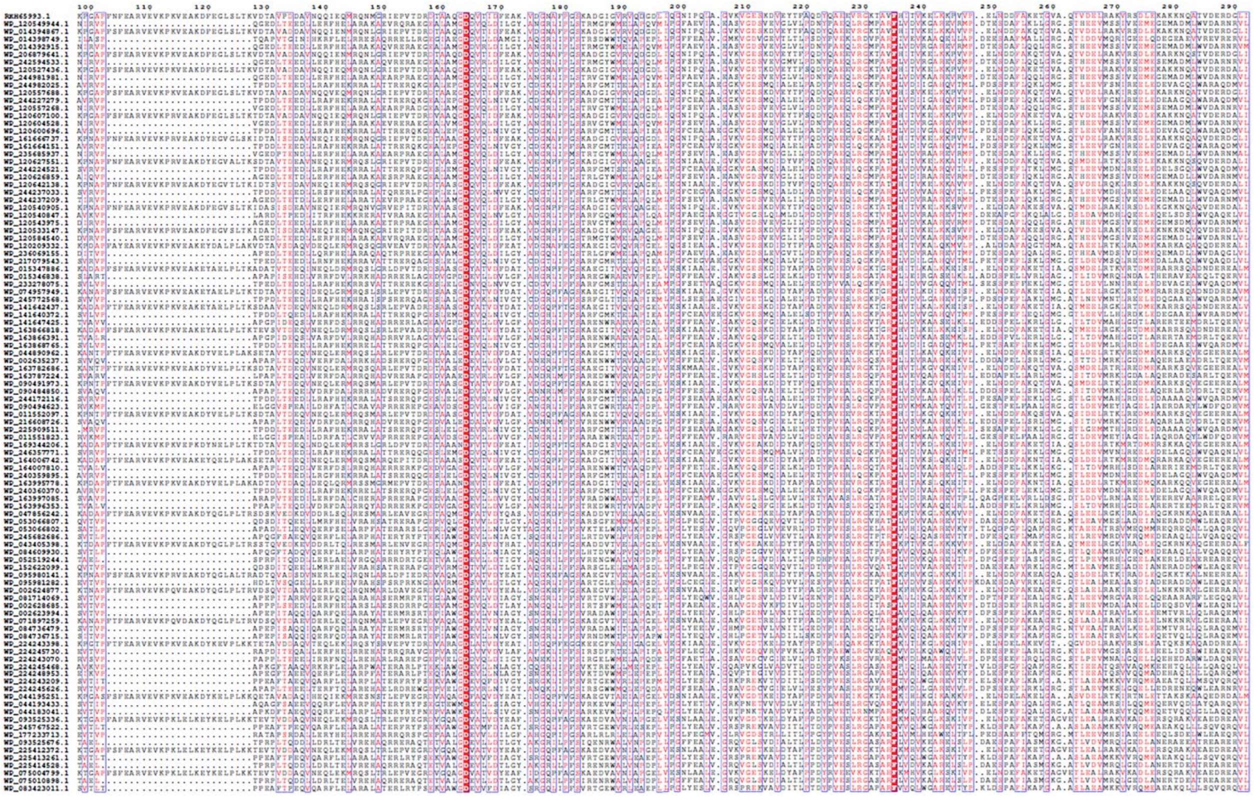

(continue with next page)

(continued)

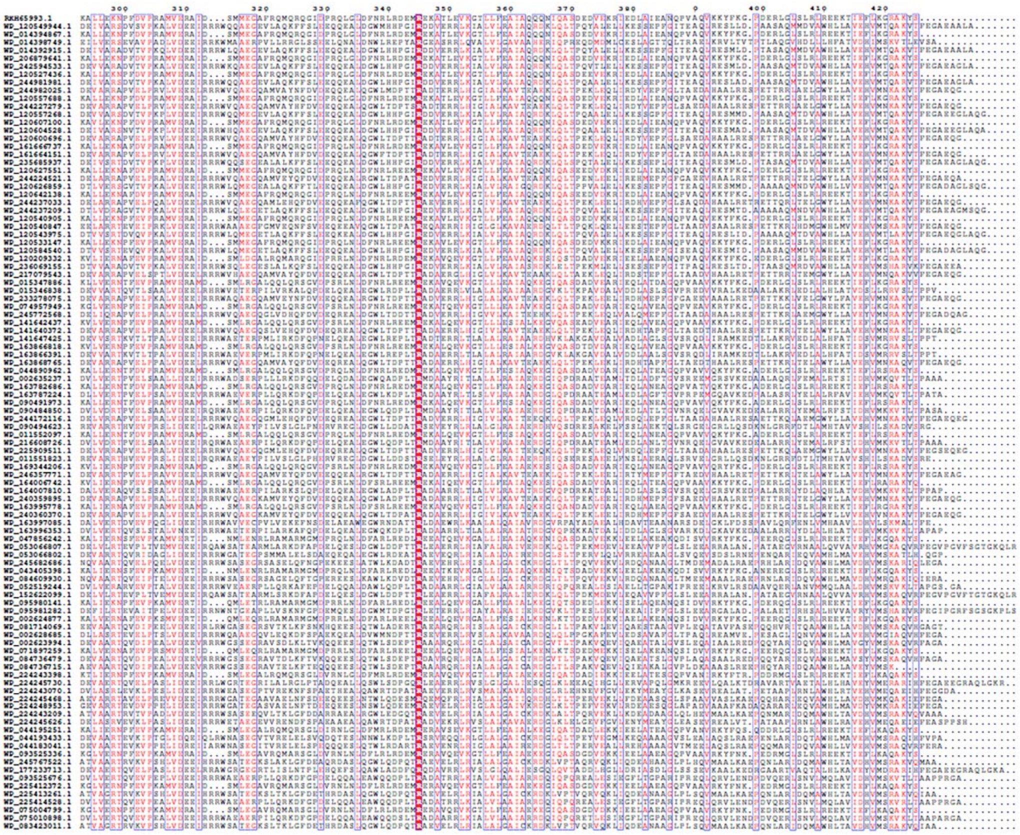

Fig. S3. Complete sequence alignment of TF homologs in 33 representative genomes of *Myxococcales* with multiple TF homologs, related to Figure S2.

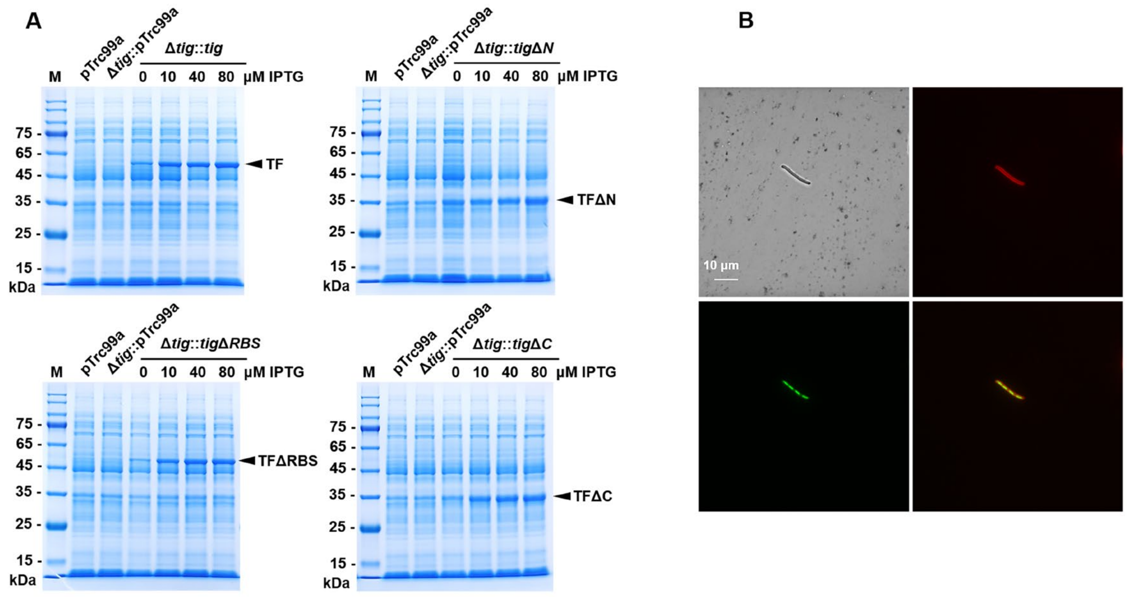

Fig. S4. Protein expression verification and polyploidy cell observation, related to Figure 2. (A) Whole-cell SDS-PAGE of *E. coli* mutants induced with different concentrations of IPTG, which indicated that the recombinant plasmids were successfully expressed. (B) Chromosome (green) and cell membrane (red) staining of TF overexpressing filamentous cells.

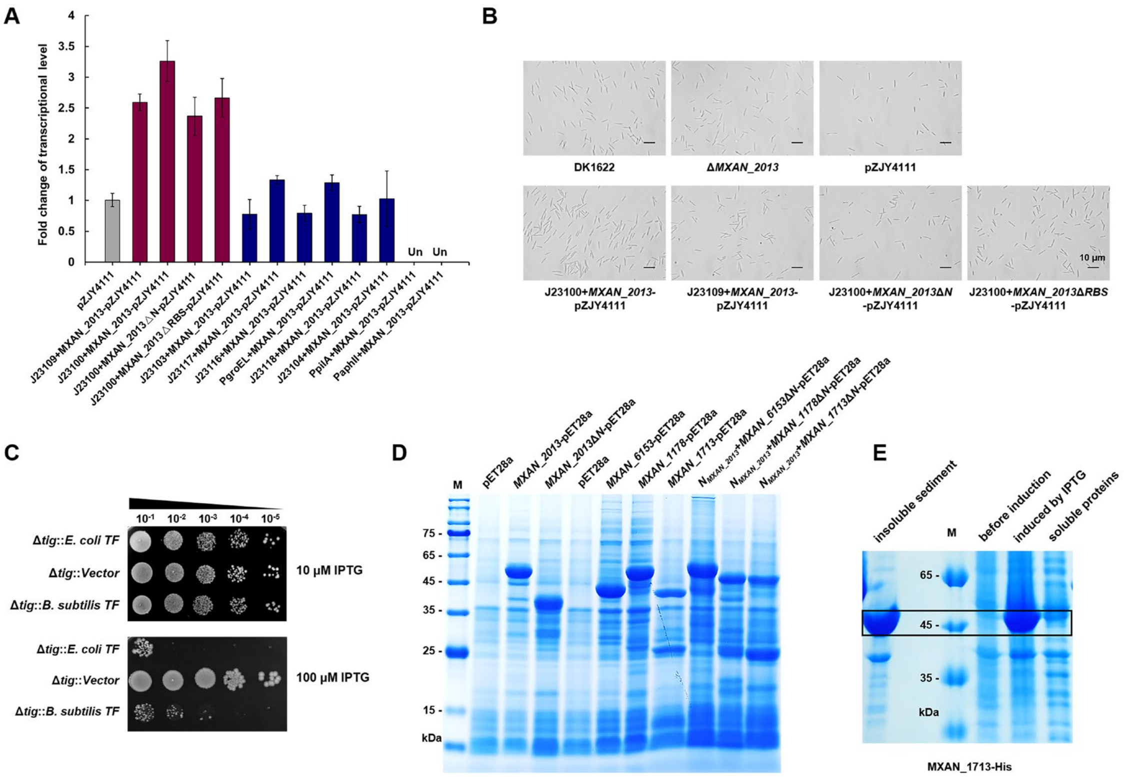

Fig. S5. Overexpression of TF homologs in *M. xanthus* DK1622 and *E. coli* BL21, related to Figure 3. (A) Fold changes in the transcriptional level of *MXAN_2013* and its variants using different promoters in *M. xanthus* DK1622. "Un" indicates that the corresponding mutant was not constructed successfully. (B) Morphological analysis of *M. xanthus* overexpression mutants under microscope. (C) Overexpression of *B. subtilis* TF in *E. coli*. (D) Whole-cell SDS-PAGE of heterogeneously expressed *E. coli* strains under 100 μM IPTG induced, indicating that the recombinant plasmids were successfully expressed. (E) Protein expression of MXAN_1713 in *E. coli* BL21. The soluble proteins and insoluble sediment were separated by centrifugation after the cells were disrupted by ultrasonication.

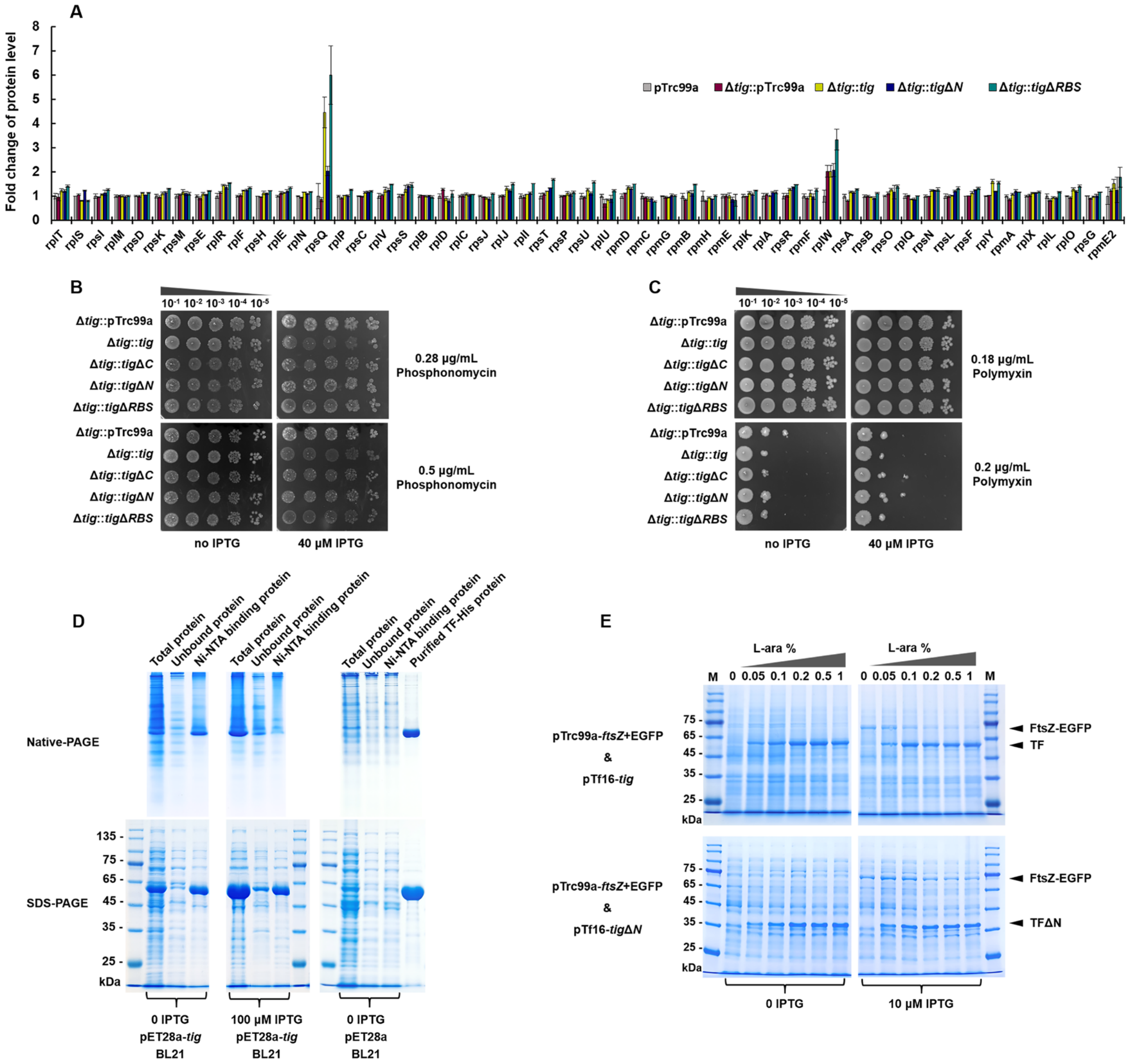

Fig. S6. Effects of TF overexpression on *E. coli* cells, related to Figure 4. (A) Fold changes of ribosome subunit proteins in different *E. coli* mutants. (B) Growth of *E. coli* mutants under phosphonomycin (which inhibits the early synthesis of bacterial cell walls) or polymyxin (which acts on bacterial cell membranes) (C) induced by 0 or 40 μM IPTG. All the LB plates contained 100 μg/ml Amp to maintain the plasmids. (D) Pull-down of the dispersive bands by TF-His. Native-PAGE and SDS-PAGE of total, unbound, and Ni-NTA binding proteins of TF-His overexpressing strain. The total protein is soluble component after ultrasonic crushing and centrifugation. (E) Whole-cell SDS-PAGE of mutants

containing pTrc99a-*ftsZ*+*egfp* with pTf16-*tig* or pTf16-*tig*Δ*N* under IPTG and serial L-arabinose concentration induction.

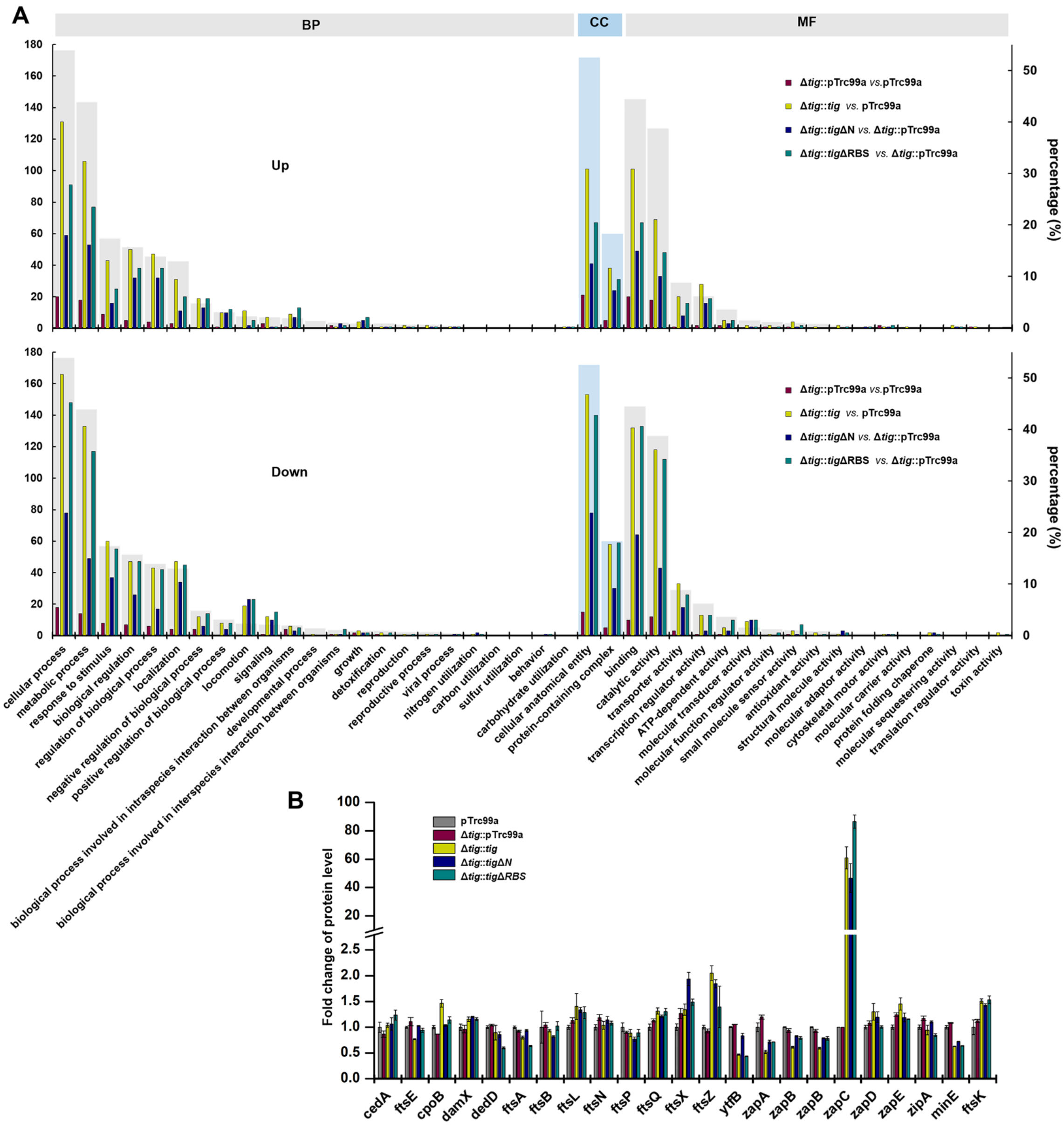

Fig. S7. Proteomic changes in the *tig*-overexpressing mutant, related to Figure 5. (A) The number of upregulated and downregulated proteins annotated by the Gene Ontology knowledgebase at the second term. BP, biological process; CC, cellular component; MF, molecular function. The background columns indicate the percentage (right y-axis) of detected proteins annotated to this term compared to the total number of detected proteins annotated by the Gene Ontology knowledgebase. (B) Fold changes in the protein levels of cell division-associated proteins in different *E. coli* mutants.

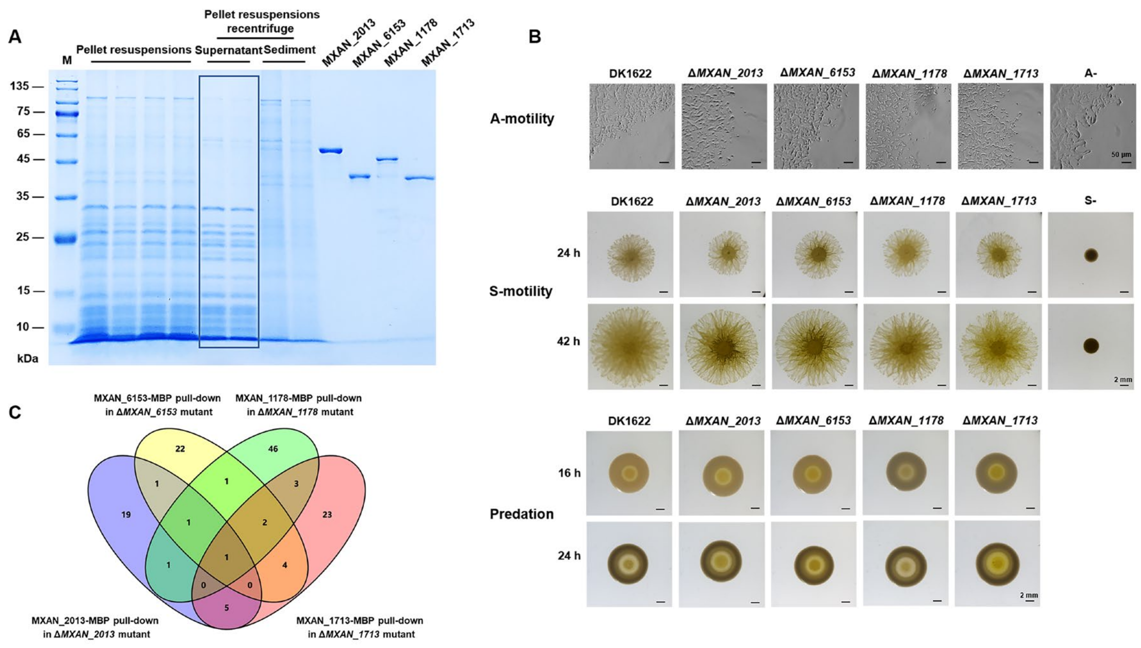

Fig. S8. Protein purification, phenotypic analysis, and potential interaction proteins of four TF homologs in *M. xanthus* DK1622, related to Figures 6 and 7. (A) SDS-PAGE of the purified ribosomes and TF proteins in *M. xanthus* DK1622. The samples in the blue frame were chosen for subsequent experiments. The sizes of the TF homologous proteins are shown on the right. The M lane represents the protein marker. (B) Social behavior analysis of the deletion mutants and wild-type strain DK1622. A-motility was characterized as the formation of single colonies distant from the edge of the swarm, and the colonies were observed with a phase contrast microscope. The strain Δ*aglZ* (A-S+) was used as a negative control. The bars in black are 50 μm. S-motility was indicated by expanding the swarm edge on 0.4% agar CTT plates and was observed with a stereoscopic microscope at 24 h and 72 h. The strain Δ*pilA* (A+S-) was used as a negative control. The white bar represents 2 mm. The predation of different strains on *E. coli* prey mats was observed via stereoscopic microscopy at 16 h and 24 h. The black bar represents 2 mm. (C) Venn diagram of the potential interacting proteins that were pulled down from the total proteins of the

corresponding deletion *M. xanthus* mutant cells by the four purified MBP labelled TF homologs.

Table S1. Number of genomes containing no or multiple TF homologs in different classes of *Proteobacteria*, *Actinobacteria*, *Firmicutes*, *Bacteroidetes*, and *Tenericutes*.

Table S2. Analysis of TF homologs in 51 representative genomes of *Myxococcales*. The black triangles mark the proteins that contain the RBS motif.

Table S3. RBS-containing TF homologs in 23 genomes with multiple RBS-containing TFs. The identities were calculated via the pair-to-pair comparisons of RBS-containing TFs in the same strain.

Table S4. Fold changes of TF interacting proteins (reference to Wayne A. Hendrickson et al., 2009) in *tig* overexpression mutant of *E. coli* (p-value <0.05).

Table S5. Potential interacting proteins that were pulled down from the total proteins of the corresponding deletion *M. xanthus* mutant cells by the four purified MBP labelled TF homologs.

Table S6. Bacterial strains and plasmids used in this study.

Table S7. List of primers used in this study.

Dataset S1 (separate file). The existence of TF homologs in prokaryotes (sheet 1). The number of TF homologs in each genome (sheet 2). Sequence analysis of TF homologs in genomes containing multiple TFs (sheet 3).

Dataset S2 (separate file). Proteomic analysis of the various *tig* overexpressed *E. coli* mutants.
